# Process Intensification Sonocatalytic Degradation of Malachite Green Wastewater over Hydrothermally Engineered M-ZLT@Co_3_O_4_ Nanocatalyst: Structure–Activity Relationships, Radical-Mediated Mechanism and Degradation Pathways

**DOI:** 10.3390/molecules31183212

**Published:** 2026-09-11

**Authors:** Murat Kıranşan

**Affiliations:** Department of Chemistry and Chemical Processing Technologies, Gümüşhane Vocational School, Gümüşhane University, 29100 Gümüşhane, Turkey; murat.kiransan@gumushane.edu.tr

**Keywords:** clay modification, degradation pathways, MG degradation, M-ZLT@Co_3_O_4_ nanocatalyst, sonocatalytic performance, textile wastewater treatment

## Abstract

This study reports the sonocatalytic degradation of malachite green (MG) in media using synthesized Co_3_O_4_ nanoparticles and a CTAB-modified zeolite supported Co_3_O_4_ hybrid nanocatalyst (M-ZLT@Co_3_O_4_). The catalysts were characterized by XRD, FT-IR, SEM-EDX, TEM, XRF, N_2_ adsorption–desorption, BET, and BJH analyses, verifying crystalline Co_3_O_4_ formation, successful immobilization on the modified zeolite support, porous architecture, morphology and elemental distribution. Under optimized conditions of 1 g L^−1^ catalyst dosage, 20 mg L^−1^ MG concentration, natural pH, and 300 W L^−1^ ultrasonic power, M-ZLT@Co_3_O_4_ achieved 99.31% ± 0.45% degradation within 120 min. Decreasing the MG concentration to 5 mg L^−1^ increased the efficiency to 99.96% ± 0.64%, confirming high activity at low pollutant loading. The degradation followed pseudo-first-order kinetics, with k_app_ values of 0.0042–0.0595 min^−1^ depending on catalyst dosage, 0.0046–0.0667 min^−1^ for initial MG concentration, and 0.0117–0.0620 min^−1^ under pH conditions. Water matrix inhibition followed distilled water (99.31%) > tap water (96.08%) > river water (88.84%) > lake water (86.90%), whereas higher ultrasonic power increased degradation from 91.98% at 200 W L^−1^ to 99.90% at 500 W L^−1^. Dye degradation followed MG (99.31%) > CR (95.78%) > BY2 (92.15%) > MV (90.24%) > AO7 (85.62%) > RB19 (82.49%). Comparative tests demonstrated performance for US/M-ZLT@Co_3_O_4_ (99.31%) compared with US/Co_3_O_4_ nanoparticles (78.48%), US/ZLT (72.86%), and US/MG alone (35.71%). The best activity was obtained at 0.8–2 g L^−1^ catalyst dosage and 5–20 mg L^−1^ MG concentration, while reusability tests showed a decrease from 99.31% to 78.82% after six cycles, indicating promising stability.

## 1. Introduction

Synthetic dyes are extensively used in textile, paper, leather, aquaculture, and pharmaceutical industries, generating large volumes of colored wastewater that pose severe environmental and public health concerns [1]. Among these, malachite green (MG), a triphenylmethane cationic dye, has attracted significant attention due to its widespread industrial application and pronounced toxicological effects. Malachite green (C_23_H_25_N_2_Cl) is commonly employed as a dyeing agent in textile processing, as a biological stain, and as an antifungal and antiparasitic agent in aquaculture [2,3]. However, its persistence, bioaccumulation potential, and transformation into toxic metabolites such as malachite green raise serious ecological and human health concerns. MG is characterized by high chemical stability, strong chromophoric properties, and resistance to biodegradation, making it recalcitrant in conventional wastewater treatment systems [4]. Even at low concentrations (≤1 mg L^−1^), MG imparts intense coloration to receiving water bodies, significantly reducing light penetration and thereby inhibiting photosynthetic activity in aquatic ecosystems. Furthermore, numerous toxicological studies have reported that MG exhibits mutagenic, teratogenic, and carcinogenic properties [5,6]. Chronic exposure has been associated with cytotoxicity, respiratory toxicity, and liver damage, while its metabolite malachite green demonstrates prolonged retention in biological tissues. Due to these risks, the use of MG in aquaculture has been banned or strictly regulated in many countries; nevertheless, its illegal and uncontrolled application continues in certain regions [7,8,9].

The discharge of MG-containing wastewater presents a significant environmental challenge, particularly in developing regions with intensive textile and aquaculture operations. Dye-laden effluents are typically characterized by high chemical oxygen demand (COD), elevated total organic carbon (TOC), complex aromatic structures, and strong resistance to conventional biological treatment due to the antimicrobial properties of MG [10]. Traditional treatment methods, including coagulation–flocculation, adsorption, ion exchange, and membrane filtration, are often insufficient for complete mineralization [11]. While adsorption using porous materials such as activated carbon or zeolites demonstrates high removal efficiency, it merely transfers contaminants from the aqueous phase to a solid matrix, necessitating regeneration or secondary disposal. Consequently, advanced oxidation processes (AOPs) have gained increasing attention as sustainable alternatives for the degradation of refractory organic pollutants [12,13,14].

In recent years, advanced oxidation processes (AOPs) have emerged as promising strategies for the degradation of recalcitrant organic pollutants such as MG. AOPs rely on the in situ generation of highly reactive oxygen species (ROS), including hydroxyl radicals (•OH), superoxide radicals (•O_2_^−^), and sulfate radicals (SO_4_•^−^), which non-selectively oxidize complex dye molecules into smaller, less toxic intermediates and eventually mineralize them into CO_2_ and H_2_O [15,16]. Among these techniques, photocatalysis, sonocatalysis, Fenton and photo-Fenton processes, and persulfate activation have demonstrated considerable potential for MG degradation [17]. Semiconductor-based photocatalysts (e.g., TiO_2_, ZnO, SnO_2_, NiO, CuO, Co_3_O_4_) have been extensively studied due to their ability to generate electron–hole pairs under light irradiation, initiating redox reactions at the catalyst surface [18]. Among AOPs, sonocatalysis has emerged as a promising and energy-efficient technology for wastewater remediation. Sonocatalysis exploits acoustic cavitation phenomena generated by ultrasonic irradiation (typically 20–100 kHz), which induce the formation, growth, and violent collapse of microbubbles in aqueous media [19]. The collapse of these cavitation bubbles generates localized “hot spots” with transient temperatures of approximately 5000 K and pressures exceeding 1000 atm, creating highly reactive conditions that promote water sonolysis and radical formation [20]. The primary sonochemical reactions responsible for ROS generation are expressed as follows in Equations (1)–(6).

*H*_2_*O + )))) (Ultrasonic irradiation) → H• + •OH* (1)

*•OH + H• → H*_2_*O* (2)

*•OH + •OH→H*_2_*O*_2_ (3)

*H*_2_*O*_2_ + *H• → H*_2_*O + •OH* (4)

•H + O_2_ → •HO_2_ (5)

*•HO*_2_ → *O*_2_•^−^ + *H^+^* (6)

In the presence of dissolved oxygen, hydroxyl radicals (•OH), hydrogen radicals (•H), hydroperoxyl radicals (•HO_2_), and superoxide radicals (O_2_•^−^) are generated, which non-selectively oxidize organic contaminants such as MG [21]. The oxidative degradation of MG can proceed through chromophore cleavage, N-demethylation, ring opening reactions, and eventual mineralization as follows Equations (7) and (8) [22].

*MG + •OH → Intermediate products* (7)

*Intermediates + •OH/O*_2_•^−^ → *CO*_2_ + *H*_2_*O + NH*_4_^+^ + *Cl*^−^ (8)

Although ultrasonic irradiation alone can degrade dyes, the process is often limited by low cavitation efficiency and insufficient radical yield. The integration of heterogeneous catalysts into ultrasonic systems significantly enhances degradation efficiency through improved ROS production, surface adsorption, and electron transfer processes [23]. The involvement of Co redox centers in ROS-mediated oxidation is further supported by previous experimental observations. Zhang et al., demonstrated, by high-resolution Co 2p XPS analysis, the coexistence of Co^2+^ and Co^3+^ species in a Co-containing catalytic system, indicating the availability of multivalent cobalt centers capable of participating in interfacial redox reactions. More importantly, in situ electrochemical measurements revealed a pronounced current response during catalytic oxidation, while cyclic voltammetry confirmed substantial redox activity of the Co-containing electrode. The increase in PMS concentration was accompanied by an enhanced current density, which was attributed to increased ROS generation and associated electron transfer during the redox process. DFT derived charge density redistribution additionally demonstrated efficient electron transfer at the Co-containing catalyst/PMS interface. Collectively, these observations provide literature-based experimental support for the participation of cobalt redox chemistry in ROS generating catalytic processes. Nevertheless, since direct monitoring of Co^2+^/Co^3+^ interconversion was not performed in the present study, the Co^3+^/Co^2+^ redox cycle is considered a plausible contributing pathway rather than a conclusively demonstrated elementary mechanism. Under ultrasonic irradiation, Co_3_O_4_ can facilitate interfacial electron excitation and promote ROS generation via the following Equations (9) and (10) [24].

*Co*^3+^ + *H*_2_*O → Co*^2+^ + *•OH + H* (9)

*Co*^2+^ + *O*_2_ *→ Co*^3+^ + *O*_2_•^−^ (10)

These redox cycles enhance the formation of hydroxyl and superoxide radicals, thereby accelerating MG degradation kinetics. Nevertheless, bare Co_3_O_4_ nanoparticles often suffer from agglomeration, limited surface area, and reduced stability in aqueous systems, which can diminish catalytic efficiency and recyclability [25]. To address these limitations, the integration of Co_3_O_4_ with surfactant modified zeolite supports has gained considerable attention. Zeolites are crystalline aluminosilicate frameworks characterized by high surface area, tunable pore structures, ion-exchange capacity, and strong structural stability [26]. Modification with cationic surfactants such as cetyltrimethylammonium bromide (CTAB) enhances the surface hydrophobicity and adsorption affinity toward organic pollutants. CTAB modification introduces positively charged quaternary ammonium groups and hydrophobic alkyl chains onto the zeolite surface, improving the electrostatic and hydrophobic interactions with MG molecules [27,28,29].

Modified CTAB-Zeolite@Co_3_O_4_ nanocatalyst system, the sonocatalytic degradation mechanism involves a synergistic interplay of adsorption, cavitation, and redox-mediated ROS formation as follows in Equations (11)–(13).

*MG_(aq)_ → MG_(ads)_* (11)

*H*_2_*O + )))) → H• + •OH* (12)

*MG_(ads)_ + •OH/O*_2_•^−^ *→ Degraded intermediates → CO*_2_ + *H*_2_*O* (13)

The porous CTAB-modified zeolite structure also acts as a cavitation nucleation site, facilitating bubble formation and enhancing mass transfer at the solid–liquid interface. Furthermore, the synergistic adsorption–oxidation coupling increases the local pollutant concentration near catalytic active sites, improving degradation kinetics typically described by pseudo-first-order or Langmuir–Hinshelwood models [30]. In this context, the present study focuses on the rational synthesis and detailed characterization of a M-ZLT@Co_3_O_4_ nanocatalyst for efficient sonocatalytic degradation of malachite green in aqueous media. The work systematically evaluates the structural, morphological, and electronic properties of the catalyst, along with degradation kinetics, reactive species identification, mineralization degree, and reusability performance [31]. This study aims to provide mechanistic insight into cobalt-based sonocatalytic systems and contribute to the development of sustainable, high-performance technologies for dye-contaminated wastewater treatment [32].

In this study, the sonocatalytic degradation of malachite green (MG), selected as a model recalcitrant organic pollutant, was systematically investigated. MG is widely recognized for its high chemical stability, intense chromophoric structure, and pronounced ecotoxicological effects, even at trace concentrations [33]. Numerous studies have demonstrated that MG exerts mutagenic, carcinogenic, and cytotoxic impacts on aquatic organisms and may pose long term risks to human health through bioaccumulation and trophic transfer. The persistence and toxicity of MG at low concentrations (<1 mg L^−1^) highlight the urgent need for efficient and sustainable degradation strategies capable of achieving not only discoloration but also substantial mineralization [34,35,36]. In this context, sonocatalytic oxidation represents a promising advanced oxidation process (AOP) because acoustic cavitation can generate high ROS under comparatively mild operating conditions. Herein, spinel-type Co_3_O_4_ nanoparticles and M-ZLT@Co_3_O_4_ nanocatalyst were synthesized through a controlled hydrothermal route and employed as heterogeneous sonocatalysts [37]. Co_3_O_4_ is a p-type transition metal oxide semiconductor with a relatively narrow and complex band-gap structure arising from electronic transitions associated with the mixed-valence Co^2+^/Co^3+^ centers within its normal spinel lattice. This electronic configuration enables Co_3_O_4_ to respond effectively to the highly energetic micro environments generated during ultrasonic cavitation. The formation, growth, and implosive collapse of cavitation bubbles produce localized hot spots, transient high pressures and temperatures, shock waves, microjets, and sonoluminescence. These effects can supply sufficient energy to promote electrons (e^−^) from occupied valence-band states to higher-energy conduction-band states, leaving positively charged holes (h^+^) behind. The resulting ultrasound-induced e^−^/h^+^ pairs can migrate toward the catalyst surface, where electrons participate in interfacial reduction reactions involving dissolved O_2_, whereas holes can oxidize surface-bound H_2_O and OH^−^ species. Consequently, reactive intermediates such as superoxide radicals (•O_2_^−^), hydroxyl radicals (•OH), and other oxygen derived species can be continuously generated during ultrasonic irradiation. Because of the relatively narrow band structure of Co_3_O_4_, repeated cavitation events occurring throughout sonication can continuously provide excitation energy, thereby sustaining charge-carrier generation and ROS-mediated oxidation. However, rapid electron–hole recombination may compete with these interfacial redox reactions; therefore, efficient spatial separation and transfer of photogenerated/sono-induced charge carriers are critical for maximizing sonocatalytic activity [38].

The hydrothermal synthesis strategy promotes controlled crystallization and homogeneous distribution of Co_3_O_4_ species over the modified zeolite framework, while surface modification of ZLT with CTAB alters the interfacial environment and enhances the affinity of the support toward MG molecules, thereby increasing pollutant enrichment in the vicinity of catalytically active sites [39]. Immobilization of Co_3_O_4_ on the CTAB-modified zeolitic matrix can further improve the accessibility and dispersion of the semiconductor phase and may suppress excessive nanoparticle aggregation. More importantly, the M-ZLT@Co_3_O_4_ interface can facilitate charge migration and decrease the probability of rapid e^−^/h^+^ recombination, allowing a greater fraction of charge carriers to participate in surface redox reactions. Simultaneously, the porous and heterogeneous surface of M-ZLT can provide additional nucleation sites for acoustic cavitation and promote mass transfer of MG, dissolved oxygen, and reactive intermediates toward the catalyst interface. Thus, the integration of narrow-band-gap Co_3_O_4_ with the modified zeolite support provides complementary effects involving enhanced pollutant enrichment, improved dispersion of active Co_3_O_4_ species, more effective utilization of ultrasound-induced charge carriers, intensified Co^2+^/Co^3+^ redox cycling, and sustained ROS generation. These mutually reinforcing processes provide a mechanistic basis for the enhanced sonocatalytic oxidation performance of the M-ZLT@Co_3_O_4_ hybrid system [40,41].

To the best of our knowledge, this study provides the first comprehensive evaluation of MG sonocatalytic degradation using pristine Co_3_O_4_ and M-ZLT@Co_3_O_4_ nanocatalysts, offering a cost-effective, stable and reusable alternative to conventional noble-metal-based systems [42]. Its novelty arises from coupling CTAB-enhanced adsorption with cobalt mediated radical generation under ultrasonic irradiation. Sonocatalytic performance was systematically assessed as a function of catalyst dosage, initial MG concentration, and solution pH to clarify kinetics and optimize degradation [43]. The process was interpreted through apparent pseudo-first-order behavior consistent with a Langmuir–Hinshelwood-type pathway. Reusability was evaluated over consecutive cycles to assess practical applicability [44], while catalyst stability and cobalt retention were considered key implementation criteria [45]. Overall, this work advances mechanistic understanding and supports sustainable cobalt-based sonocatalytic treatment of dye containing wastewater under realistic environmental treatment conditions [46].

## 2. Results and Discussion

### 2.1. Characterization of Co_3_O_4_ NPs and M-ZLT@Co_3_O_4_ Nanocatalyst

The Co_3_O_4_ nanoparticles, and the M-ZLT@Co_3_O_4_ hybrid nanocatalyst used as catalysts in the sonocatalytic degradation of malachite green dye waste were synthesized as illustrated in Figure 14a. Subsequently, the synthesized Co_3_O_4_, and M-ZLT@Co_3_O_4_ nanocatalysts, together with the pure ZLT clay employed as the supporting material, were characterized using various analytical techniques.

#### 2.1.1. XRD Analysis

**Figure 1:**
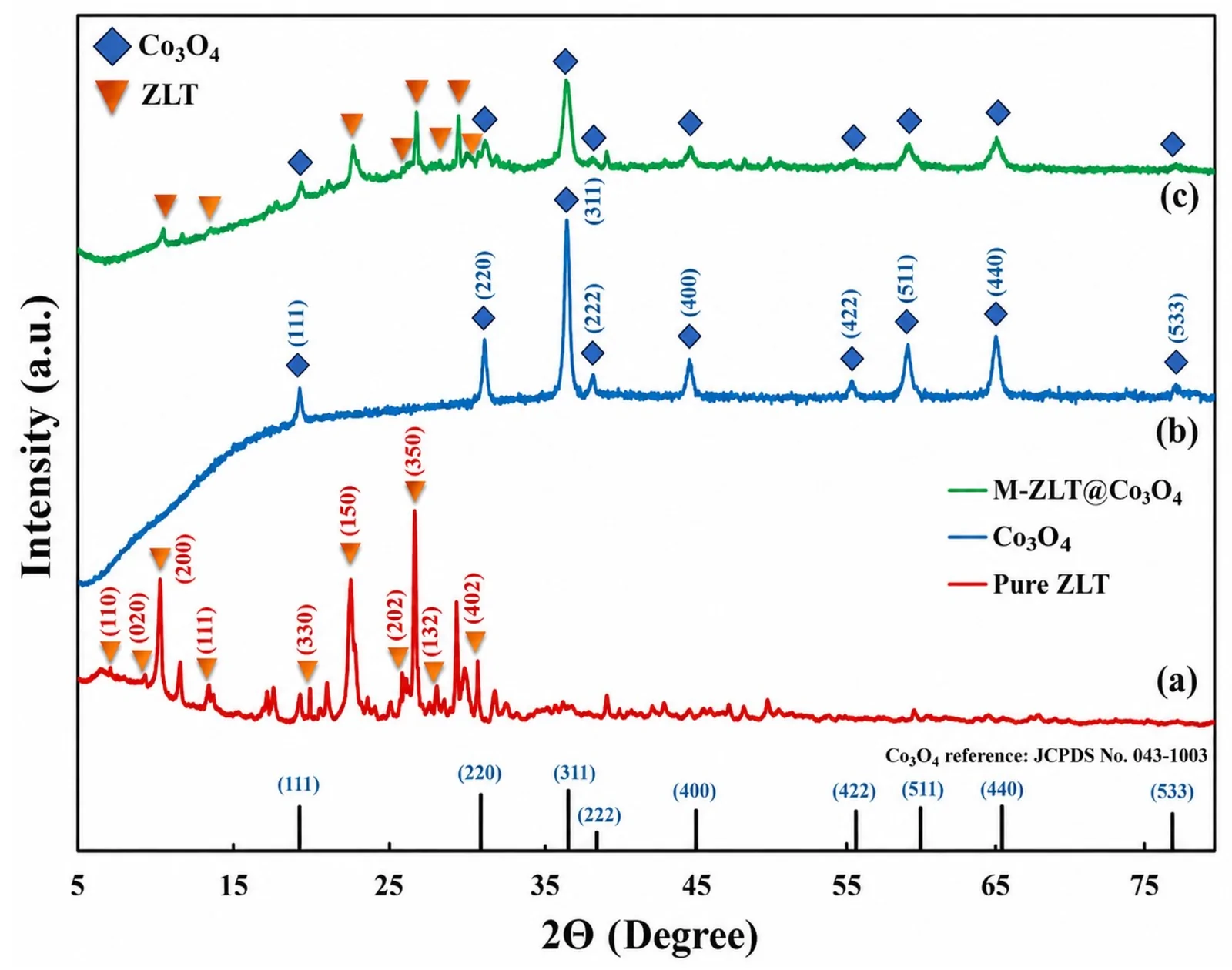
XRD diffractograms of the synthesized catalysts: (a) Pure ZLT, (b) Co_3_O_4_ nanoparticles, (c) M-ZLT@Co_3_O_4_ hybrid nanocatalyst.

The XRD patterns of pure ZLT, Co_3_O_4_ nanoparticles, and the M-ZLT@Co_3_O_4_ nanocatalyst are presented in Figure 1. The diffraction pattern of the pure zeolite clay exhibited several characteristic reflections at 2θ values of 6.5°, 8.7°, 9.8°, 13.3°, 19.7°, 22.4°, 25.7°, 26.4°, 27.7°, and 30.8°, which can be indexed to the (110), (020), (200), (111), (330), (150), (202), (350), (132), and (402) crystallographic planes, respectively (Figure 1 (a)). These diffraction peaks are in good agreement with the standard zeolite reference pattern JCPDS No. 43-0171, confirming the crystalline aluminosilicate framework of the ZLT support [47].

The presence of intense reflections, particularly in the low- and medium-angle regions, indicates that the zeolite clay possesses a well-defined crystalline structure. In addition, the relatively sharp zeolite peaks suggest that the framework ordering of the natural zeolitic clay was preserved before modification and Co_3_O_4_ loading [48].

For the pure Co_3_O_4_ nanoparticles, the XRD pattern displayed typical diffraction peaks at approximately 19.0°, 31.3°, 36.9°, 38.6°, 44.9°, 55.8°, 59.4°, 65.2°, and 77.4°, which are assigned to the (111), (220), (311), (222), (400), (422), (511), (440), and (533) crystal planes, respectively (Figure 1 (b)). These reflections match well with the cubic spinel phase of Co_3_O_4_ with the Fd3m space group, according to JCPDS No. 043-1003 [49]. The strong diffraction peak located around 36.9°, corresponding to the (311) plane, represents the most intense reflection of spinel Co_3_O_4_ and confirms the successful formation of crystalline Co_3_O_4_ nanoparticles. The absence of additional diffraction peaks related to cobalt oxide impurities such as CoO, Co(OH)_2_, or other cobalt-containing phases indicates that the synthesized Co_3_O_4_ nanoparticles were obtained with high phase purity within the detection limits of XRD [50]. In the XRD pattern of the M-ZLT@Co_3_O_4_ nanocatalyst, diffraction peaks belonging to both the zeolite clay support and cubic spinel Co_3_O_4_ phase can be clearly observed (Figure 1 (c)). The characteristic reflections of Co_3_O_4_, especially those corresponding to the (220), (311), (400), (422), (511), (440), and (533) planes, are retained in the composite structure, demonstrating that the crystalline spinel phase of Co_3_O_4_ was successfully formed and immobilized on the modified zeolite surface. At the same time, the characteristic diffraction peaks of ZLT are still detectable, particularly in the lower 2θ region, indicating that the zeolite framework was not destroyed during the modification and composite synthesis process [51].

Compared with pure ZLT, the diffraction peaks of zeolite in the M-ZLT@Co_3_O_4_ nanocatalyst show relatively reduced intensity and partial overlap with Co_3_O_4_ reflections. This behavior can be attributed to the deposition of Co_3_O_4_ nanoparticles on the external surface and pores of the modified zeolite clay, which partially covers the zeolite crystalline domains and decreases their relative diffraction contribution. Moreover, the incorporation of Co_3_O_4_ and surface modification of ZLT may cause slight changes in peak intensity due to interfacial interactions, particle dispersion, and the reduced relative amount of zeolite in the final composite [52]. However, no significant shift or disappearance of the main zeolite peaks is observed, suggesting that the crystalline structure of ZLT remains largely preserved after Co_3_O_4_ loading. The coexistence of the characteristic peaks of both components in the composite pattern provides strong evidence for the successful fabrication of the M-ZLT@Co_3_O_4_ hybrid nanocatalyst (Figure 1 (c)). The preservation of the cubic spinel Co_3_O_4_ phase together with the zeolitic framework is particularly important for catalytic applications because the zeolite support can provide high surface area, adsorption sites, and improved dispersion of Co_3_O_4_ nanoparticles, while Co_3_O_4_ acts as the active sonocatalytic phase [53]. Therefore, the XRD results confirm not only the successful synthesis of phase pure Co_3_O_4_ nanoparticles but also their effective integration with modified zeolite clay without severe structural deterioration of the support. As a result, the XRD analysis verifies that the M-ZLT@Co_3_O_4_ nanocatalyst consists of crystalline cubic spinel Co_3_O_4_ nanoparticles anchored onto the modified zeolitic clay matrix [54].

The crystallite sizes of the zeolite clay, Co_3_O_4_ and M-ZLT@Co_3_O_4_ nanocatalysts were quantitatively evaluated using the Debye–Scherrer equation. This is expressed as follows Equation (14).

(14) d =(kλ/βcosθ)

where k is the Debye–Scherrer constant (0.89), λ is the X-ray wavelength (0.15406 nm), β is the full width at half maximum (FWHM) of the diffraction peak in radians, and θ represents the Bragg diffraction angle. The crystallite size of the zeolite phase was estimated from the diffraction peak located at 2θ = 26.4°, which is associated with the crystalline structure of zeolite, using the Debye–Scherrer equation. Based on this calculation, the average crystallite size of zeolite was determined to be approximately 32.6 nm. Similarly, the crystallite sizes of Co_3_O_4_ in both the synthesized Co_3_O_4_ nanoparticles and the M-ZLT@Co_3_O_4_ nanocatalyst were calculated using the same equation [55]. For pure Co_3_O_4_, the characteristic diffraction peak at 2θ = 36.9° was selected, and the crystallite size was found to be approximately 12.8 nm. In the case of the M-ZLT@Co_3_O_4_ nanocatalyst, the diffraction peak observed at 2θ = 37.05° was used for the calculation, yielding an estimated crystallite size of about 16.4 nm. These results indicate that Co_3_O_4_ nanoparticles were successfully incorporated into the modified zeolite matrix while maintaining their nanoscale crystalline nature [56].

#### 2.1.2. FTIR Analysis

**Figure 2:**
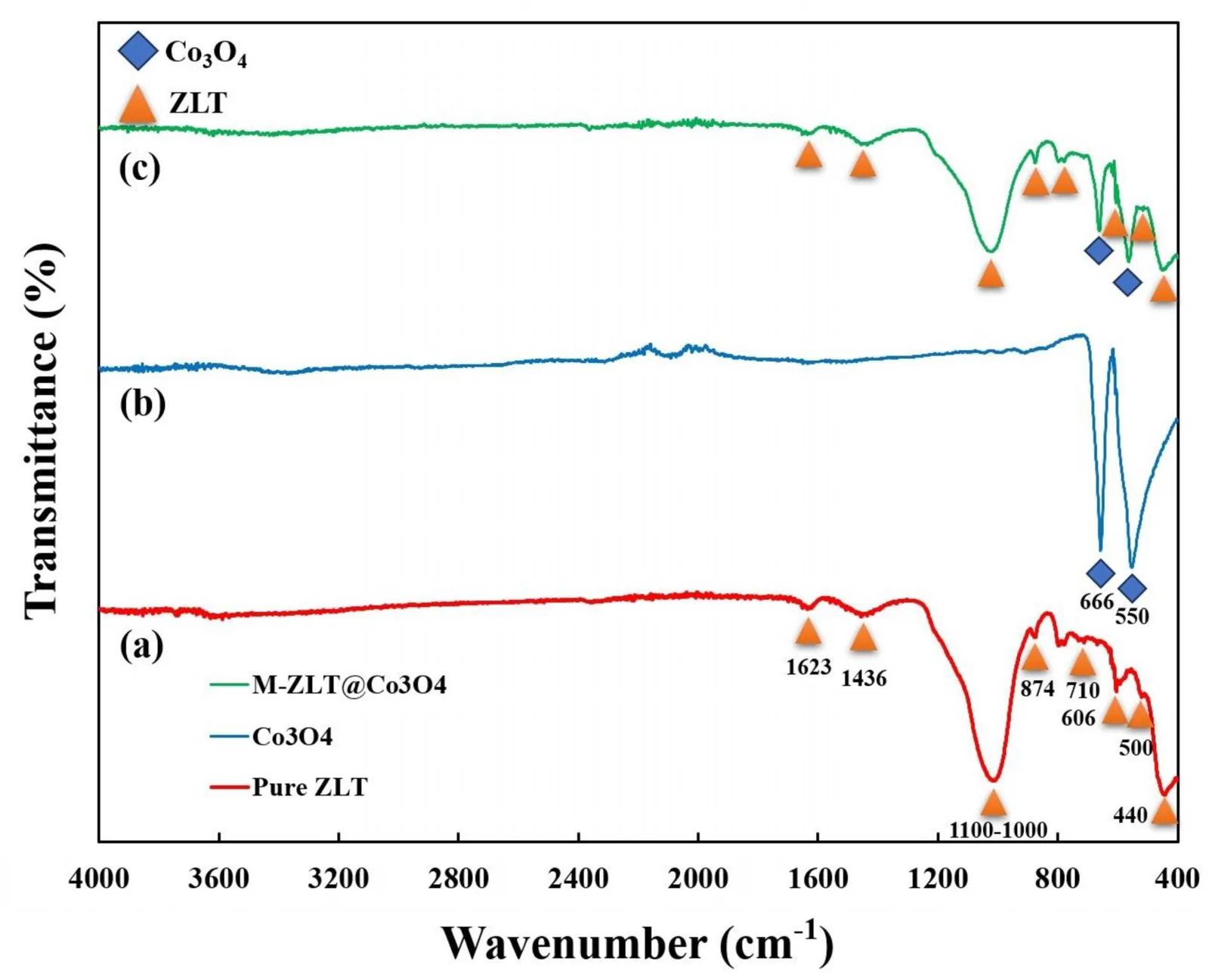
FT-IR spectra of the synthesized catalysts, showing: (a) Pure ZLT, (b) Co_3_O_4_ nanoparticles (c) M-ZLT@Co_3_O_4_ hybrid nanocatalyst.

The FT-IR spectra of pure ZLT, Co_3_O_4_ nanoparticles, and the M-ZLT@Co_3_O_4_ nanocatalyst are shown in Figure 2. The spectrum of pure zeolite clay exhibits the typical vibrational features of an aluminosilicate framework. The absorption bands observed in the low-wavenumber region of approximately 440–500 cm^−1^ can be assigned to bending vibrations associated with tetrahedral Al–O bonds. The strong and broad band located in the 1000–1100 cm^−1^ region is characteristic of asymmetric stretching vibrations of the Si–O–Si and Si–O–Al framework units, which are the main structural components of zeolitic materials (Figure 2 (a)). This intense band confirms the presence of the ordered aluminosilicate network in the ZLT structure. In addition, several characteristic bands are observed in the 500–1000 cm^−1^ region, which are attributed to internal vibrations of Si–O and Al–O bonds in the zeolite framework. The band around 500 cm^−1^ can be related to external tetrahedral vibrations of Si–O and Al–O units. The peaks located at 606 cm^−1^ and 710 cm^−1^ correspond to symmetric stretching vibrations of Si–O–Si and Si–O–Al, respectively (Figure 2 (a)). Furthermore, the bands detected at 1623 cm^−1^ and 1436 cm^−1^ are associated with C–O–C stretching vibrations, while the band observed around 3740 cm^−1^ is attributed to surface hydroxyl groups or –OH stretching vibrations. These results confirm the presence of hydroxylated surface groups and the characteristic aluminosilicate framework of the zeolite clay [57].

For the pure Co_3_O_4_ nanoparticles, the FT-IR spectrum shows two distinct absorption bands at approximately 666 cm^−1^ and 550 cm^−1^ (Figure 2 (b)). These bands are assigned to the stretching vibrations of Co–O bonds in the spinel Co_3_O_4_ structure. The presence of these two characteristic bands indicates the formation of cobalt oxide with spinel-type metal–oxygen coordination. In particular, the Co–O vibration bands in this region are consistent with the tetrahedral and octahedral cobalt–oxygen environments of Co_3_O_4_, confirming the successful synthesis of Co_3_O_4_ nanoparticles [58]. In the FT-IR spectrum of the M-ZLT@Co_3_O_4_ nanocatalyst, the characteristic bands of both ZLT and Co_3_O_4_ are observed, demonstrating the successful formation of the composite structure. The aluminosilicate-related bands of ZLT, especially those in the 1000–1100 cm^−1^, 606 cm^−1^, 710 cm^−1^, 500 cm^−1^, and 440 cm^−1^ regions, are retained in the spectrum of M-ZLT@Co_3_O_4_ (Figure 2 (c)). This indicates that the zeolitic framework was largely preserved after modification and Co_3_O_4_ incorporation. At the same time, the Co–O vibration bands originating from Co_3_O_4_ are also present in the low-wavenumber region, confirming the immobilization of Co_3_O_4_ nanoparticles onto the modified zeolite surface [59]. Compared with pure ZLT, the M-ZLT@Co_3_O_4_ spectrum shows changes in band intensity and partial broadening of some characteristic zeolite vibrations. These changes can be attributed to the interaction between Co_3_O_4_ nanoparticles and the surface functional groups of modified ZLT. The incorporation of Co_3_O_4_ may partially cover the zeolite surface and affect the local bonding environment of Si–O, Al–O, and surface –OH groups. Such spectral changes support the existence of interfacial interactions between the modified zeolite matrix and Co_3_O_4_ nanoparticles rather than a simple physical mixture [60].

In conclusion, the FT-IR results confirm that the M-ZLT@Co_3_O_4_ nanocatalyst contains the characteristic functional groups and framework vibrations of zeolite together with the typical Co–O stretching bands of Co_3_O_4_. The preservation of the zeolite framework bands, together with the appearance of Co_3_O_4_ related vibrations, provides strong evidence for the successful synthesis of the hybrid nanocatalyst. These findings are consistent with the XRD results and indicate that Co_3_O_4_ nanoparticles were effectively incorporated into the modified zeolitic clay structure without significant destruction of the aluminosilicate framework [61,62].

#### 2.1.3. SEM/EDX Analysis

**Figure 3:**
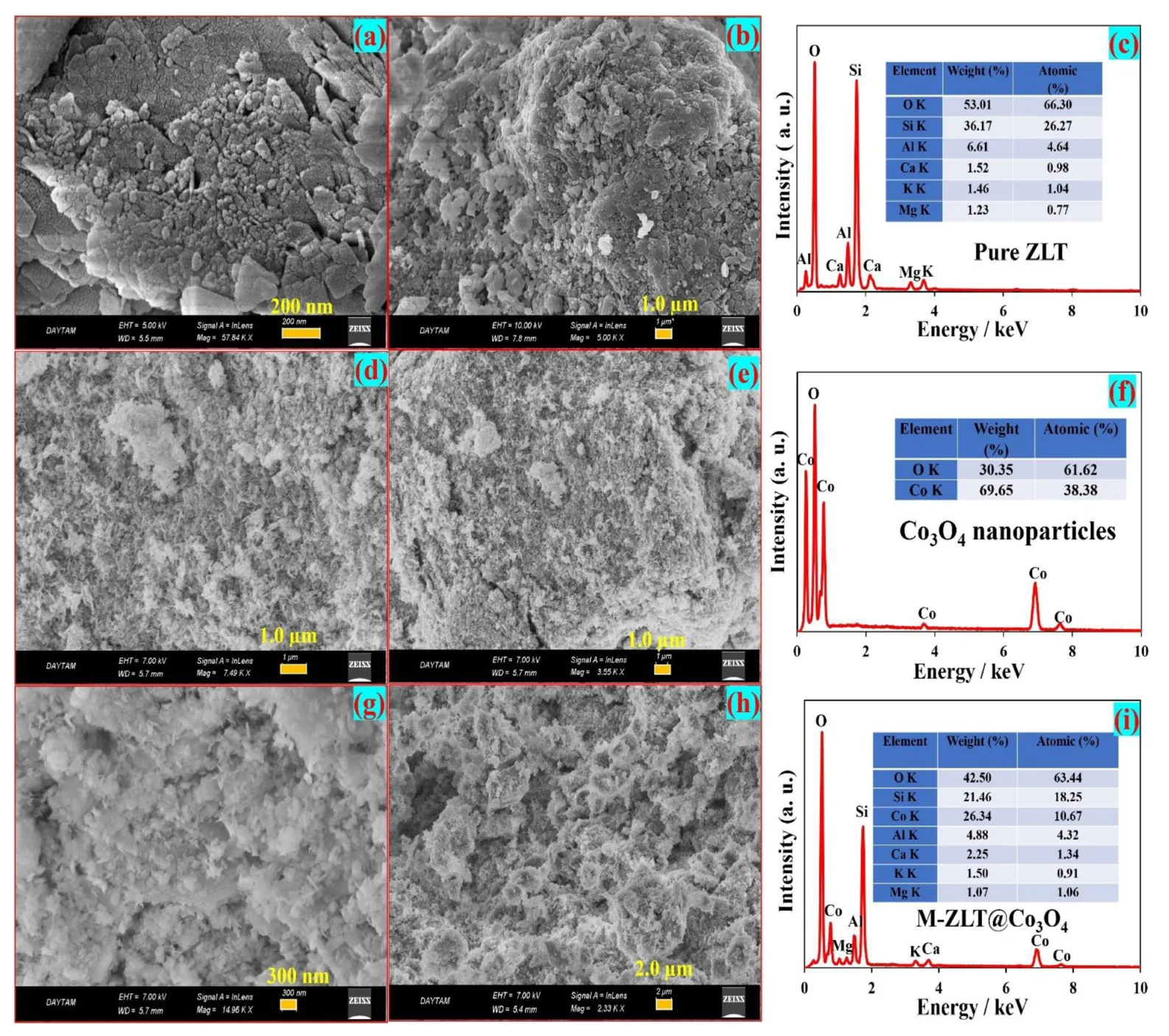
SEM images of (**a**,**b**) pure ZLT, (**d**,**e**) Co_3_O_4_ nanoparticles (**g**,**h**) M-ZLT@Co_3_O_4_ hybrid nanocatalyst and EDX spectra of (**c**) pure ZLT, (**f**) Co_3_O_4_ nanoparticles, (**i**) M-ZLT@Co_3_O_4_ hybrid nanocatalyst.

The surface morphology and elemental composition of pure ZLT, Co_3_O_4_ nanoparticles, and the M-ZLT@Co_3_O_4_ hybrid nanocatalyst were investigated by SEM and EDX analyses, and the corresponding images and spectra are presented in Figure 3. The SEM micrographs of pure ZLT shown in Figure 3a,b reveal a typical layered, plate-like, and irregular morphology, which is characteristic of natural zeolitic clay minerals. The ZLT surface consists of stacked sheet-like structures, fractured edges, cracks, and heterogeneous domains. These morphological features indicate the presence of a naturally rough and defect rich aluminosilicate framework. Such a heterogeneous surface is highly favorable for surface modification and nanoparticle immobilization because cracks, cavities, and interlayer spaces can act as anchoring sites for metal oxide particles. In addition, the rough morphology of ZLT may contribute to pollutant adsorption by increasing the available contact area between the catalyst surface and the aqueous solution. At higher magnification, the SEM image of pure ZLT further confirms that the surface is not smooth or compact but rather consists of irregular aggregates and microstructural discontinuities. These structural characteristics are important for catalytic applications because the presence of surface defects and interparticle voids can facilitate mass transfer and enhance the interaction between pollutant molecules and active catalytic sites. Therefore, pure ZLT can serve not only as a support material but also as an adsorptive matrix for improving the dispersion and accessibility of the active Co_3_O_4_ phase in the hybrid nanocatalyst. The EDX spectrum of pure ZLT in Figure 3c confirms the presence of O, Si, Al, Ca, K, and Mg elements. The dominant elemental components are oxygen and silicon, with weight percentages of 53.01 wt.% and 36.17 wt.%, respectively. Aluminum is also detected at 6.61 wt.%, confirming the aluminosilicate nature of the zeolitic clay framework. The minor amounts of Ca, K, and Mg, corresponding to 1.52, 1.46, and 1.23 wt.%, respectively, can be attributed to exchangeable extra-framework cations naturally present in the zeolite structure. The strong O, Si, and Al signals are consistent with the chemical structure of zeolite-based clay minerals composed mainly of Si–O and Al–O tetrahedral units. Thus, the EDX results support the SEM observations and verify the mineralogical composition of the ZLT support [63].

The SEM images of the synthesized Co_3_O_4_ nanoparticles shown in Figure 3d,e display a significantly different morphology compared with pure ZLT. The Co_3_O_4_ sample exhibits a highly aggregated and rough surface composed of small granular particles. These nanoparticles appear to be assembled into irregular clusters, which may be associated with the high surface energy of nanosized cobalt oxide particles. Such agglomeration is commonly observed in metal oxide nanoparticles prepared through hydrothermal and calcination processes. Although particle aggregation is present, the rough and porous like texture of Co_3_O_4_ can provide numerous active sites for catalytic reactions. In sonocatalytic systems, this type of morphology is particularly beneficial because rough surfaces can promote acoustic cavitation, increase interfacial interactions, and accelerate the generation of ROS. The EDX spectrum of Co_3_O_4_ nanoparticles in Figure 3f shows only cobalt and oxygen peaks, confirming the successful formation of cobalt oxide without detectable impurity elements. The elemental composition consists of 69.65 wt.% Co and 30.35 wt.% O, which is consistent with the expected composition of cobalt oxide nanoparticles. The strong Co signals observed in the EDX spectrum further confirm that cobalt is the major metallic component of the synthesized nanoparticles [64].

After the incorporation of Co_3_O_4_ nanoparticles onto the modified zeolite clay support, the SEM images of the M-ZLT@Co_3_O_4_ hybrid nanocatalyst shown in Figure 3g,h reveal a distinct morphological transformation. Compared with pure ZLT, the hybrid material exhibits a more compact, rough, porous, and sponge like surface structure. The original layered morphology of ZLT appears to be partially covered by fine Co_3_O_4_ nanoparticles, suggesting the successful deposition and immobilization of the cobalt oxide phase on the modified zeolite surface. The formation of this hybrid structure indicates that ZLT acts as a suitable support matrix for dispersing Co_3_O_4_ nanoparticles and reducing excessive particle aggregation. The SEM images also show that the surface of M-ZLT@Co_3_O_4_ contains numerous irregular pores, cavities, and interconnected rough domains. This morphology is highly advantageous for sonocatalytic degradation because it can enhance the adsorption of MG molecules near the active Co_3_O_4_ sites and improve the contact between the pollutant, catalyst, and reactive radical species. The EDX spectrum of M-ZLT@Co_3_O_4_ in Figure 3i provides strong evidence for the successful formation of the hybrid nanocatalyst. The spectrum contains the characteristic elements of both ZLT and Co_3_O_4_, including O, Si, Al, Ca, K, Mg, and Co. The presence of 26.34 wt.% Co in the hybrid sample confirms that Co_3_O_4_ nanoparticles were effectively incorporated into the modified ZLT matrix. Meanwhile, the detection of Si, Al, Ca, K, and Mg demonstrates that the zeolitic clay framework was retained after the hybridization process. The oxygen content of the hybrid material is 42.50 wt.%, while Si and Al are detected at 21.46 wt.% and 4.88 wt.%, respectively. These values indicate that the aluminosilicate structure remains present, although its relative elemental contribution decreases due to the successful loading of the cobalt oxide phase [65].

Compared with pure ZLT, the relative decrease in Si and Al percentages in the M-ZLT@Co_3_O_4_ hybrid nanocatalyst can be attributed to the partial coverage of the ZLT surface by Co_3_O_4_ nanoparticles. This observation is consistent with the SEM results, where the surface of the hybrid material appears more densely covered and morphologically modified. In addition, the simultaneous detection of Co together with the characteristic zeolitic elements confirms the coexistence of the metal oxide phase and the clay-based support within the same hybrid structure. Therefore, the EDX results validate the successful anchoring of Co_3_O_4_ nanoparticles onto the modified-ZLT surface.

As a result, the SEM and EDX analyses clearly demonstrate that the M-ZLT@Co_3_O_4_ hybrid nanocatalyst was successfully synthesized. Pure ZLT exhibits a layered, cracked, and heterogeneous aluminosilicate morphology, while Co_3_O_4_ nanoparticles show an aggregated granular structure. After hybrid formation, the resulting M-ZLT@Co_3_O_4_ nanocatalyst displays a rough, porous, and highly textured surface, indicating effective interaction between Co_3_O_4_ nanoparticles and the modified-ZLT support. The coexistence of Co, O, Si, Al, Ca, K, and Mg in the EDX spectrum confirms the successful combination of the cobalt oxide active phase with the zeolite clay matrix [66].

#### 2.1.4. TEM Analysis

**Figure 4:**
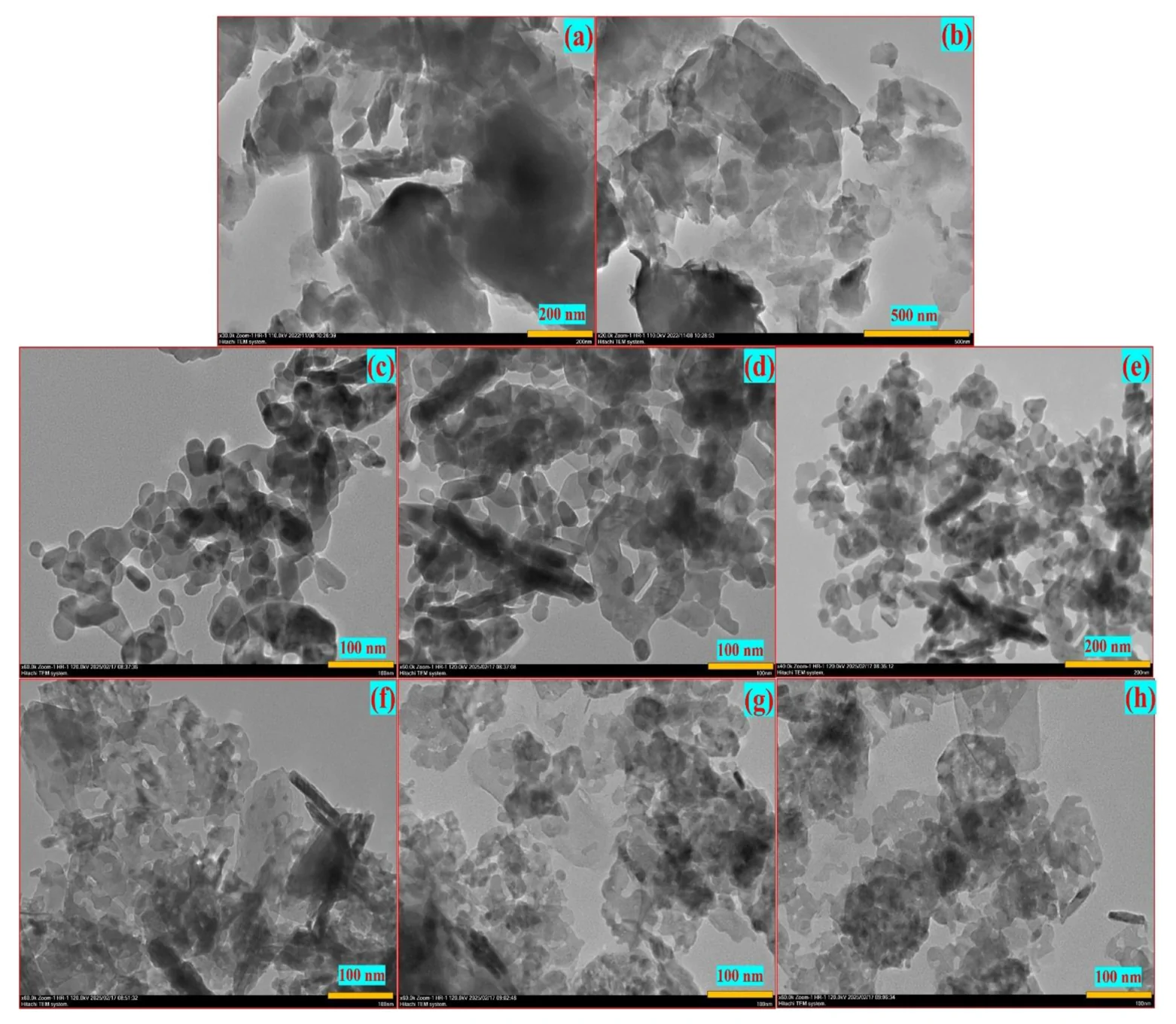
TEM images of (**a**,**b**) Pure ZLT, (**c**–**e**) Co_3_O_4_ nanoparticles, (**f**–**h**) M-ZLT@Co_3_O_4_ hybrid nanocatalyst.

The TEM images presented in Figure 4 provide further insight into the internal morphology, particle dispersion, and structural organization of pure ZLT, Co_3_O_4_ nanoparticles, and the M-ZLT@Co_3_O_4_ hybrid nanocatalyst. The TEM micrographs of pure ZLT shown in Figure 4a,b reveal irregular, layered, and sheet-like structures with semi-transparent regions. This morphology is consistent with the typical lamellar architecture of zeolite clay minerals. The presence of thin, plate-like domains and overlapping layers indicates that ZLT possesses a heterogeneous aluminosilicate framework with variable thickness. The darker regions observed in the images can be attributed to thicker stacked layers or agglomerated clay domains, whereas the lighter transparent areas correspond to thinner nanosheet-like regions. Such a layered morphology is beneficial for catalytic applications because it provides a suitable support surface for the immobilization of metal oxide nanoparticles [66,67].

The TEM images of Co_3_O_4_ nanoparticles in Figure 4c–e show that the synthesized cobalt oxide particles exhibit nanoscale dimensions with irregular spherical, oval, and short rod-like morphologies. The nanoparticles are not completely isolated but are partially agglomerated, which is commonly observed for metal oxide nanoparticles due to their high surface energy and strong interparticle interactions. Nevertheless, the particles remain distinguishable in several regions, indicating the successful formation of nanosized Co_3_O_4_ structures. The coexistence of small rounded particles and elongated rod-like particles suggests anisotropic growth during the hydrothermal synthesis and subsequent thermal treatment. For the M-ZLT@Co_3_O_4_ hybrid nanocatalyst, the TEM images in Figure 4f–h reveal a clear structural combination of the layered ZLT matrix and Co_3_O_4_ nanoparticles. Compared with pure ZLT, the hybrid material exhibits a more complex morphology, in which dark Co_3_O_4_ nanoparticles are distributed over or embedded within the lighter zeolitic clay sheets. The contrast difference between the ZLT support and Co_3_O_4_ particles confirms the coexistence of both components in the hybrid structure. The darker regions are associated with the higher electron density of cobalt oxide, while the lighter and more transparent regions correspond to the aluminosilicate ZLT support. The TEM observations indicate that the ZLT support plays an important role in dispersing the Co_3_O_4_ nanoparticles and limiting excessive particle aggregation [67]. Although some local agglomeration is visible, Co_3_O_4_ particles appear to be successfully anchored onto the ZLT surface rather than forming completely separated bulk clusters. This intimate interfacial contact between Co_3_O_4_ and modified ZLT is highly advantageous for catalytic performance because it can improve surface accessibility, enhance pollutant adsorption around active sites, and facilitate interfacial radical transfer during sonocatalytic reactions.

As a result, the TEM images strongly support the successful fabrication of the M-ZLT@Co_3_O_4_ hybrid nanocatalyst. Pure ZLT displays a layered and sheet-like clay morphology, while Co_3_O_4_ nanoparticles exhibit nanosized granular and partially rod-like structures. After hybridization, Co_3_O_4_ nanoparticles are effectively distributed over the modified ZLT matrix, producing a heterogeneous and rough composite architecture. This structural configuration is expected to improve sonocatalytic activity by increasing the number of accessible active sites, enhancing catalyst–pollutant interaction, reducing severe Co_3_O_4_ agglomeration, and promoting ROS generation under ultrasonic irradiation. Therefore, the TEM findings are in good agreement with the SEM and EDS results and confirm the successful formation of the M-ZLT@Co_3_O_4_ hybrid nanocatalyst [68].

#### 2.1.5. Nitrogen Adsorption–Desorption Isotherms, BJH Pore Size Distribution, and BET Textural Properties

**Figure 5:**
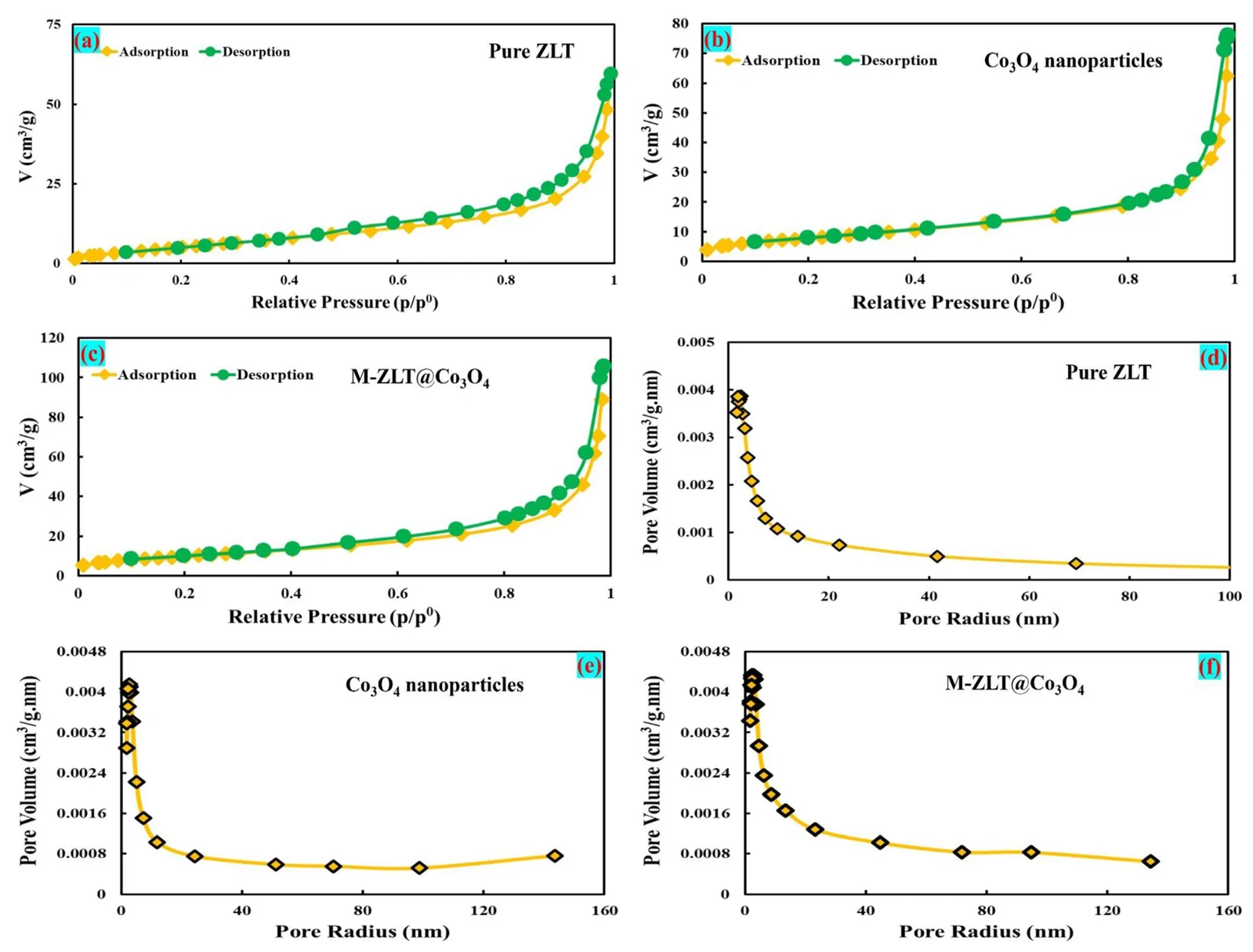
Nitrogen adsorption–desorption isotherms of (**a**) pure ZLT, (**b**) Co_3_O_4_ nanoparticles, (**c**) M-ZLT@Co_3_O_4_ hybrid nanocatalyst and BJH pore size distribution of the samples (**d**) pure ZLT, (**e**) Co_3_O_4_ nanoparticles, (**f**) M-ZLT@Co_3_O_4_ hybrid nanocatalyst.

The nitrogen adsorption–desorption isotherms and BJH pore size distribution curves of pure ZLT, Co_3_O_4_ nanoparticles, and M-ZLT@Co_3_O_4_ hybrid nanocatalyst are presented in Figure 5, while the corresponding surface area and porosity parameters are summarized in Table 1. The N_2_ adsorption–desorption isotherm of pure ZLT in Figure 5a shows a gradual increase in the adsorbed nitrogen volume with increasing relative pressure. According to the IUPAC classification, this behavior can be assigned to a Type II isotherm with a hysteresis loop, indicating multilayer physical adsorption on a heterogeneous surface. The presence of hysteresis confirms capillary condensation within the pore network of ZLT. In particular, the observed hysteresis loop may be associated with an H4-type hysteresis loop, which is generally attributed to narrow slit like pores and the coexistence of micropores and mesopores. This result is consistent with the layered and sheet-like morphology of ZLT observed in SEM and TEM images. The slit-shaped pores likely originate from the stacking of aluminosilicate sheets, interlayer spaces, and irregular voids between clay particles [69].

In order to Co_3_O_4_ nanoparticles in Figure 5b, the adsorption–desorption isotherm also exhibits a clear hysteresis loop, especially at high relative pressure values. This indicates the presence of mesopores and interparticle voids formed between aggregated Co_3_O_4_ nanoparticles. The sharp increase in nitrogen adsorption near p/p^0^ = 0.9–1.0 suggests capillary condensation in larger mesopores or textural pores. This behavior agrees well with SEM and TEM observations, where Co_3_O_4_ nanoparticles showed aggregated granular and partially rod-like structures. The porosity of Co_3_O_4_ is therefore mainly associated with interparticle spaces rather than highly ordered internal pores. The isotherm of the M-ZLT@Co_3_O_4_ hybrid nanocatalyst in Figure 5c displays a more pronounced nitrogen uptake compared with pure ZLT and Co_3_O_4_ nanoparticles, particularly at high relative pressures. This indicates that the hybridization of modified ZLT with Co_3_O_4_ nanoparticles improves the porous architecture of the material. The increase in adsorption capacity can be attributed to the formation of additional interfacial voids, surface defects, and mesoporous channels between the ZLT support and Co_3_O_4_ nanoparticles. The hysteresis loop observed for M-ZLT@Co_3_O_4_ confirms the existence of capillary condensation in mesoporous structures, while the steep rise at high p/p^0^ indicates the presence of larger interparticle pores [69,70].

The BJH pore size distribution curves in Figure 5d–f further support these conclusions. For pure ZLT, the pore size distribution is mainly concentrated in the low pore-radius region, confirming the presence of narrow pores. The extended tail toward larger pore radius indicates that ZLT also contains mesopores and larger interparticle voids. This broad distribution is typical for natural zeolite clay materials with layered and heterogeneous structures. In order to Co_3_O_4_ nanoparticles, the BJH curve shows a broad pore size distribution extending to higher pore radius. This suggests that the nanoparticle aggregates generate mesoporous and macropore like interparticle spaces. The BJH pore size distribution of M-ZLT@Co_3_O_4_ confirms the formation of a more developed hierarchical pore structure. The hybrid nanocatalyst contains narrow mesopores together with wider pores formed by the interaction between Co_3_O_4_ nanoparticles and the modified ZLT matrix [70].

According to the textural parameters given in Table 1, the incorporation of Co_3_O_4_ nanoparticles onto modified ZLT causes a clear change in the BET surface area, total pore volume, and average pore size of the resulting hybrid nanocatalyst. In general, the improved surface area and pore volume of M-ZLT@Co_3_O_4_ compared with the individual components indicate that the hybridization process generates additional accessible pores and interfacial cavities (Table 1). The increase in pore volume is particularly important because it provides more diffusion pathways for MG molecules and allows better penetration of the pollutant into the catalyst structure. Moreover, the developed mesoporous structure can reduce diffusion limitations during sonocatalytic treatment. From a sonocatalytic point of view, the textural properties of M-ZLT@Co_3_O_4_ are highly favorable. First, the porous ZLT support can adsorb MG molecules and concentrate them near the active Co_3_O_4_ sites. Second, the mesoporous channels and interparticle voids facilitate rapid mass transfer between the solution phase and the catalyst surface. Third, the rough and porous surface can act as nucleation centers for acoustic cavitation bubbles under ultrasonic irradiation. The collapse of these bubbles near the catalyst surface generates localized hot spots and reactive oxygen species such as •OH and •O_2_^−^, which accelerate the oxidative degradation of MG. Therefore, the enhanced pore structure of M-ZLT@Co_3_O_4_ contributes directly to its improved sonocatalytic performance [71].

#### 2.1.6. XRF Analysis

The bulk chemical compositions of pure ZLT and the M-ZLT@Co_3_O_4_ hybrid nanocatalyst were determined by XRF analysis, and the results are summarized in Table 2. In order to pure ZLT, the dominant oxide components are SiO_2_ and Al_2_O_3_, with contents of 71.86% and 13.22%, respectively. This result confirms the aluminosilicate nature of the zeolite clay structure, in which SiO_4_ and AlO_4_ tetrahedral units constitute the main framework. The high SiO_2_ content indicates that the ZLT sample has a silica-rich structure, while the presence of Al_2_O_3_ confirms the typical zeolitic aluminosilicate framework. The calculated SiO_2_/Al_2_O_3_ ratio of pure ZLT is approximately 5.44, suggesting a relatively silica-rich zeolite structure, which may contribute to its chemical stability and adsorption behavior. In addition to SiO_2_ and Al_2_O_3_, pure ZLT contains minor amounts of CaO, K_2_O, MgO, Fe_2_O_3_, and Na_2_O, with values of 2.76%, 2.61%, 2.28%, 1.41%, and 0.18%, respectively (Table 2). These oxides are commonly associated with naturally occurring exchangeable cations present in zeolite clay minerals. In particular, Ca^2+^, K^+^, Mg^2+^, Na^+^, and Fe-containing species may be located in the channels, cavities, or interlayer regions of the zeolite structure [72].

After the incorporation of Co_3_O_4_ nanoparticles, the chemical composition of the M-ZLT@Co_3_O_4_ hybrid nanocatalyst changes significantly. The most remarkable difference is the appearance of a high Co_3_O_4_ content of 49.26%, which clearly confirms the successful loading of cobalt oxide onto the modified ZLT support. This high cobalt oxide fraction indicates that Co_3_O_4_ constitutes the major active phase in the hybrid nanocatalyst. Meanwhile, the continued presence of SiO_2_ and Al_2_O_3_, with contents of 37.34% and 5.88%, respectively, demonstrates that the aluminosilicate framework of ZLT is still retained after the modification and hybridization process. Compared with pure ZLT, the relative amounts of SiO_2_ and Al_2_O_3_ decrease markedly in the M-ZLT@Co_3_O_4_ hybrid nanocatalyst. This decrease should not be interpreted as the destruction of the zeolitic framework; rather, it mainly results from the dilution effect caused by the introduction of a large amount of Co_3_O_4_ into the composite structure. Since Co_3_O_4_ contributes nearly half of the total oxide composition, the relative percentages of the original ZLT components naturally decrease. The preservation of SiO_2_, Al_2_O_3_, CaO, K_2_O, MgO, Fe_2_O_3_, and Na_2_O in the hybrid sample confirms that the ZLT matrix remains an integral part of the final material. The M-ZLT@Co_3_O_4_ hybrid nanocatalyst also contains CaO, K_2_O, MgO, Fe_2_O_3_, and Na_2_O at lower percentages of 1.35%, 1.21%, 1.19%, 0.97%, and 0.06%, respectively (Table 2). The decrease in these oxide contents compared with pure ZLT is also consistent with the successful incorporation of Co_3_O_4_. These residual oxides indicate that the mineralogical features of ZLT are preserved in the hybrid structure. The XRF results are in good agreement with the SEM–EDX findings. While EDX analysis confirmed the local presence of Co, Si, Al, O, Ca, K, and Mg on the surface of M-ZLT@Co_3_O_4_, XRF analysis further verifies the bulk incorporation of Co_3_O_4_ into the hybrid nanocatalyst. Therefore, the combination of EDX and XRF results provides strong compositional evidence for the successful construction of the M-ZLT@Co_3_O_4_ hybrid system [73].

### 2.2. Removal of Malachite Green (MG) by Sonocatalytic Degradation Process

In this section, the optimum operational conditions for the sonocatalytic degradation process were systematically evaluated. To this end, the principal parameters influencing degradation efficiency were comprehensively examined, including the initial concentration of MG, the dosage of the M-ZLT@Co_3_O_4_ nanocatalyst, the effect of the water matrix, effect of the ultrasonic power, solution pH, and the comparative performance of different catalysts toward MG sonocatalytic degradation. Moreover, the effects of organic and inorganic radical scavengers were investigated to clarify the dominant reactive species governing the degradation process. The sonocatalytic degradation behavior of various dye pollutants in the presence of the M-ZLT@Co_3_O_4_ nanocatalyst was also examined. Finally, the reusability and stability of the M-ZLT@Co_3_O_4_ nanocatalyst were evaluated over successive degradation cycles to assess its long time catalytic durability.

#### 2.2.1. Effect of M-ZLT@Co_3_O_4_ Nanocatalyst Concentration

**Figure 6:**
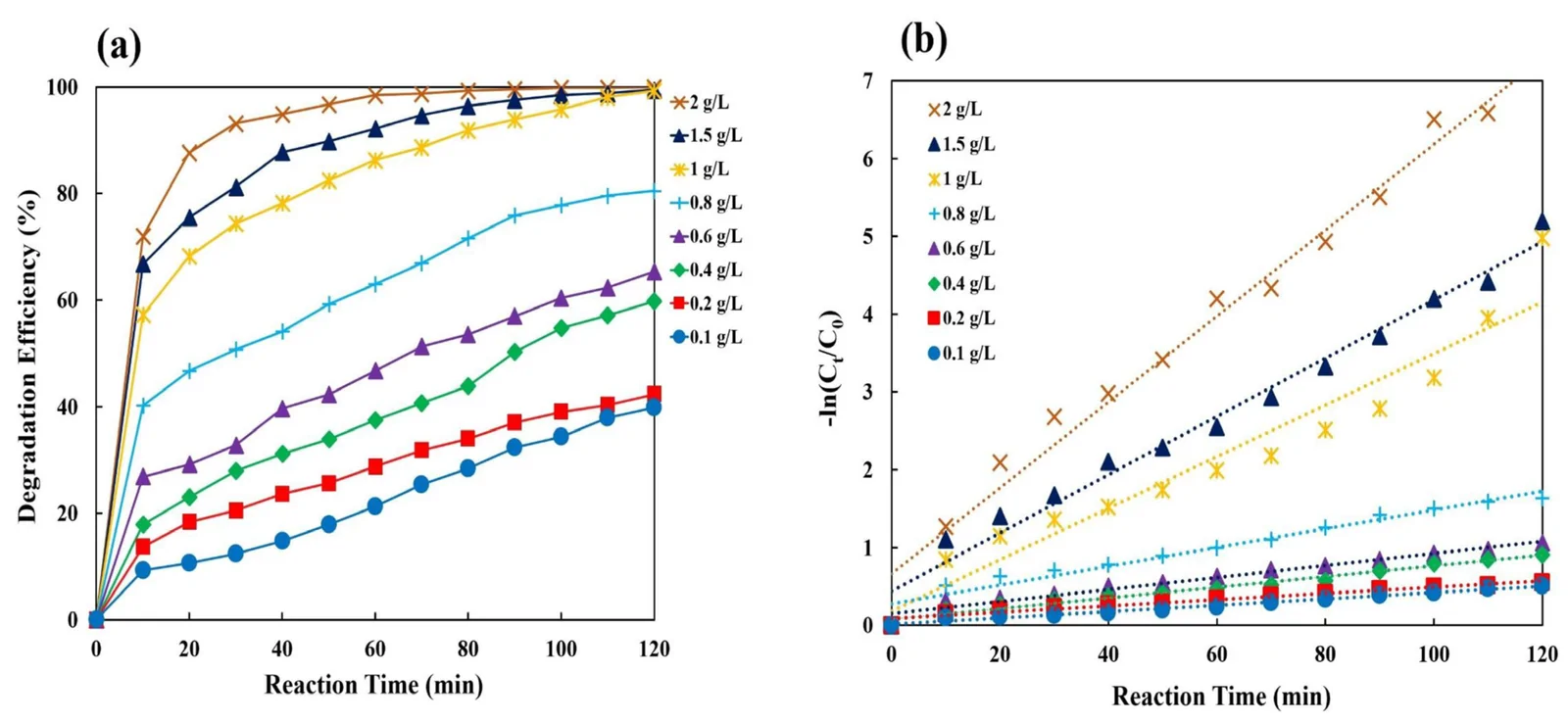
(**a**) The effect of M-ZLT@Co_3_O_4_ nanocatalyst dosage on the degradation of MG the sonocatalytic degradation process, (**b**) Apparent pseudo-first-order reaction kinetics (Experimental Conditions: Initial MG concentration: 20 mg L^−1^, Ultrasonic power: 300 W L^−1^, pH for MG: Natural pH, Reaction time: 120 min).

The effect of M-ZLT@Co_3_O_4_ nanocatalyst dosage on the sonocatalytic degradation of malachite green (MG) was systematically evaluated under the following experimental conditions, initial MG concentration of 20 mg L^−1^, ultrasonic power of 300 W L^−1^, natural pH, and a reaction time of 120 min. As clearly illustrated in Figure 6a, the degradation efficiency of MG was strongly dependent on the catalyst loading. As the dosage of M-ZLT@Co_3_O_4_ increased from 0.1 to 2.0 g L^−1^, the sonocatalytic degradation efficiency increased markedly from 39.86% to 99.92%. In particular, the degradation efficiencies were determined as 39.86%, 42.30%, 59.72%, 65.31%, 80.49%, 99.31%, 99.44%, and 99.92% for catalyst dosages of 0.1, 0.2, 0.4, 0.6, 0.8, 1.0, 1.5, and 2.0 g L^−1^, respectively (Figure 6a).

This pronounced enhancement in degradation performance with increasing catalyst dosage can be attributed to the greater availability of active surface sites and the increased formation of reactive oxygen species during ultrasonic irradiation. At low catalyst concentrations, the limited number of accessible active centers restricts the interaction between MG molecules and the catalyst surface, thereby suppressing the generation of oxidative radicals such as •OH and O_2_•^−^ [74]. As the catalyst dosage increased, more active sites became available for pollutant adsorption and radical-mediated oxidation, which significantly accelerated the degradation process. Moreover, the larger catalyst surface area likely promoted more efficient cavitation-related interfacial reactions, thereby enhancing the overall sonocatalytic activity of the system.

However, although the degradation efficiency continued to increase beyond 1.0 g L^−1^, the improvement became marginal, indicating that the system approached a saturation regime. The difference in degradation efficiency between 1.0 g L^−1^ (99.31%), 1.5 g L^−1^ (99.44%), and 2.0 g L^−1^ (99.92%) was very small, suggesting that further addition of catalyst did not yield a proportional significant enhancement (Figure 6a). This behavior may be associated with particle agglomeration, reduced effective ultrasonic wave penetration, partial shielding of active sites, and mass-transfer limitations at excessive catalyst loadings. Therefore, from both practical and economic perspectives, 1.0 g L^−1^ can be considered the optimum M-ZLT@Co_3_O_4_ dosage for the sonocatalytic degradation of MG, as it provided nearly complete removal within 120 min without the need for excess catalyst usage [75].

The kinetic behavior of malachite green (MG) degradation in the presence of different dosages of M-ZLT@Co_3_O_4_ nanocatalyst was evaluated using the pseudo-first-order kinetic model, commonly expressed as Equation (47) where k_app_ is the apparent rate constant. As shown in Figure 6b, the obtained results showed that the pseudo-first-order model described the sonocatalytic degradation process satisfactorily over the entire catalyst dosage range, as evidenced by the relatively high correlation coefficients (R^2^ = 0.9325–0.9873). These high R^2^ values indicate a strong linear relationship between −ln (C_t_/C_0_) and reaction time, confirming that the degradation of MG followed pseudo-first-order kinetics under the applied experimental conditions (Figure 6b) [76].

Moreover, the apparent rate constant (k_app_) increased markedly with increasing M-ZLT@Co_3_O_4_ dosage, rising from 0.0042 min^−1^ at 0.1 g L^−1^ to 0.0595 min^−1^ at 2.0 g L^−1^ (Table 3a). At low catalyst loadings (0.1–0.8 g L^−1^), the k_app_ values remained relatively low, ranging from 0.0042 to 0.0136 min^−1^, indicating limited degradation kinetics due to the insufficient number of accessible active sites and the relatively low generation of reactive oxidative species under ultrasonic irradiation. In contrast, a substantial increase in k_app_ was observed at catalyst dosages of 1.0 g L^−1^ and above, with values of 0.0414, 0.0432, and 0.0595 min^−1^ at 1.0, 1.5, and 2.0 g L^−1^, respectively (Table 3a). This behavior clearly demonstrates that increasing the catalyst amount significantly accelerated the sonocatalytic degradation rate of MG by enhancing the availability of catalytic active centers and promoting more effective radical-mediated oxidation pathways [77].

In conclusion, the kinetic findings confirm that catalyst dosage is a key operational parameter controlling the sonocatalytic performance of the M-ZLT@Co_3_O_4_ catalyst. The progressive increase in k_app_ with catalyst loading, together with the consistently high R^2^ values, demonstrates that the pseudo-first-order kinetic model is appropriate for describing the degradation of MG in this system. These results further suggest that higher catalyst dosages improve the reaction rate primarily by increasing surface-active sites, strengthening catalyst pollutant interactions, and facilitating the formation of reactive species responsible for MG oxidation [78].

#### 2.2.2. Effect of Initial MG Concentration

**Figure 7:**
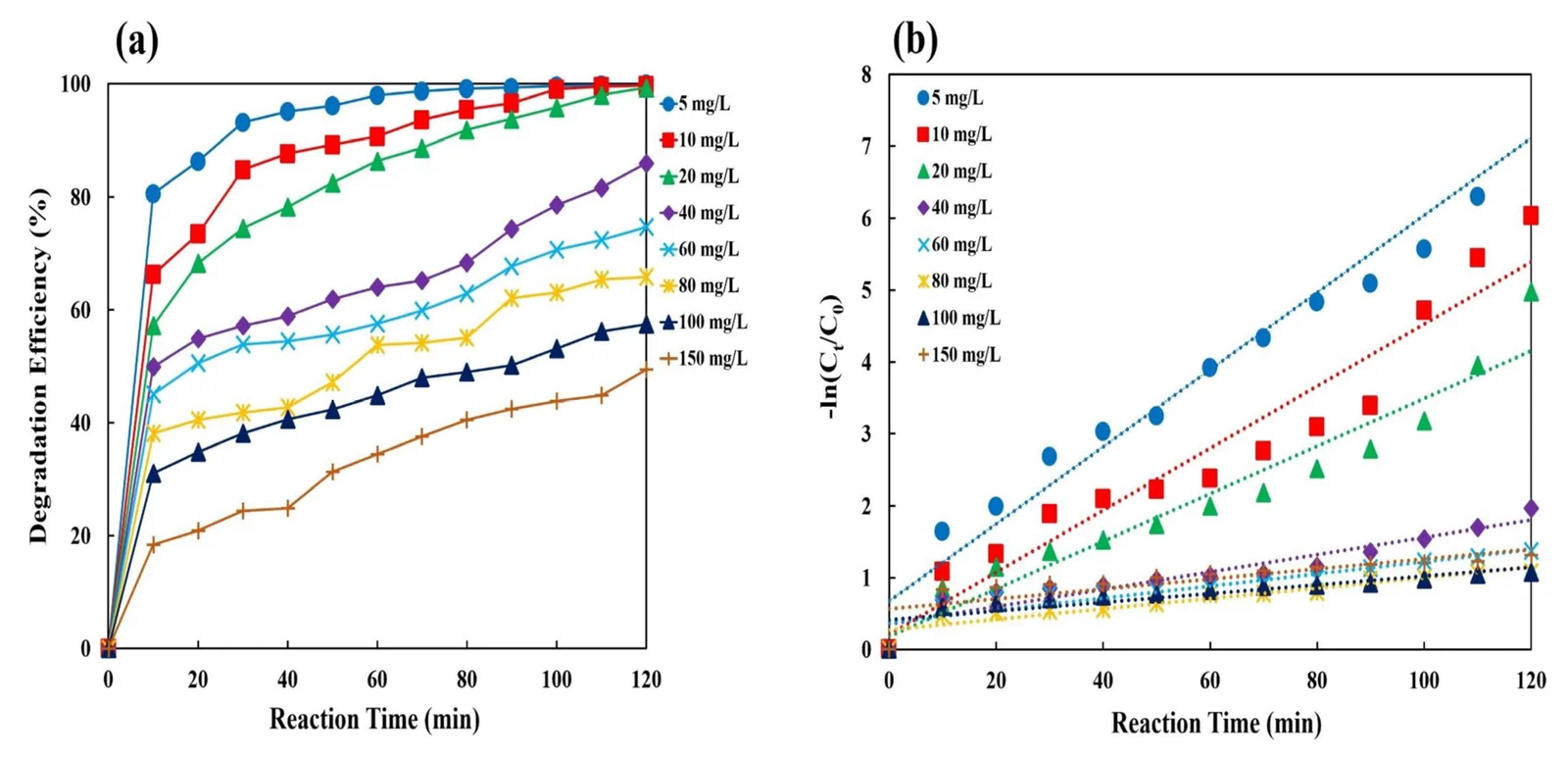
(**a**) Effect of initial dye concentration on the degradation of MG by sonocatalytic degradation process. (**b**) Apparent pseudo-first-order reaction kinetics (Experimental Conditions: Catalyst dosage: 1.0 g L^−1^, Ultrasonic power: 300 W L^−1^, pH for MG: Natural pH, Reaction time: 120 min).

The effect of the initial malachite green (MG) concentration on the sonocatalytic degradation performance of the M-ZLT@Co_3_O_4_ nanocatalyst was systematically investigated under the experimental conditions of 1.0 g L^−1^ catalyst dosage, 300 W L^−1^ ultrasonic power, natural pH, and 120 min reaction time. As shown in Figure 7a the results clearly demonstrated that the degradation efficiency of MG was strongly influenced by its initial concentration. At relatively low MG concentrations of 5, 10, and 20 mg L^−1^, the sonocatalytic degradation efficiencies were remarkably high, reaching 99.96%, 99.75%, and 99.31%, respectively. However, as the initial dye concentration increased further, the degradation efficiency gradually declined to 85.96% at 40 mg L^−1^, 74.62% at 60 mg L^−1^, 65.79% at 80 mg L^−1^, 57.43% at 100 mg L^−1^, and 49.46% at 150 mg L^−1^ (Figure 7a).

This decreasing trend can be attributed to several factors associated with the increased pollutant load in the reaction medium. At lower initial MG concentrations, the number of dye molecules present in the solution is relatively small compared with the available active sites on the catalyst surface and the amount of reactive oxygen species generated during ultrasonic irradiation. Under these conditions, MG molecules can be more effectively adsorbed and oxidized, resulting in near-complete degradation [79]. In contrast, at higher initial concentrations, the number of MG molecules increases substantially, leading to intensified competition for the limited active sites and reactive radical species such as hydroxyl radicals (•OH) and superoxide radicals (•O_2_^−^). As a result, the oxidizing species generated in the system become insufficient to completely mineralize or degrade the larger amount of dye molecules present. In addition, higher MG concentrations may reduce the efficiency of ultrasonic cavitation and interfacial radical attack by increasing solution opacity and the accumulation of degradation intermediates, both of which can hinder mass transfer and suppress effective catalyst–pollutant interactions. The adsorption of excessive dye molecules and intermediate products on the catalyst surface may also partially block active centers, thereby lowering the overall sonocatalytic performance. Therefore, the results indicate that lower initial MG concentrations favor more efficient sonocatalytic degradation, while increasing dye concentration imposes a significant kinetic and oxidative burden on the M-ZLT@Co_3_O_4_ catalyst [80].

As shown in Figure 7b, the results demonstrated that the pseudo-first-order model adequately described the sonocatalytic degradation of MG over the investigated concentration range, as confirmed by the relatively high correlation coefficients (R^2^ = 0.9325–0.9597). A pronounced inverse relationship was observed between the initial MG concentration and the apparent rate constant. As the initial dye concentration increased from 5 to 150 mg L^−1^, the k_app_ value progressively decreased from 0.0667 to 0.0046 min^−1^. Specifically, the k_app_ values were determined as 0.0667, 0.0502, 0.0414, 0.0163, 0.0114, 0.0089, 0.0067, and 0.0046 min^−1^ for initial MG concentrations of 5, 10, 20, 40, 60, 80, 100, and 150 mg L^−1^, respectively (Table 3b). This clear decline in kinetic rate with increasing MG concentration indicates that the sonocatalytic degradation process became progressively slower as the pollutant loading increased [81].

This behavior can be attributed to the fixed amount of catalyst and the limited generation of reactive oxygen species under constant ultrasonic power. At lower MG concentrations, the number of dye molecules in solution is relatively low compared with the available active sites and oxidative radical species, allowing rapid adsorption and efficient radical-mediated degradation. In contrast, at higher initial MG concentrations, the catalyst surface becomes increasingly occupied by dye molecules, while the available hydroxyl and superoxide radicals become insufficient to effectively oxidize the larger number of pollutant molecules [82]. In addition, the accumulation of dye molecules and degradation intermediates near the catalyst surface may hinder mass transfer and reduce the accessibility of active centers, thereby lowering the overall reaction rate. Overall, the kinetic results (Figure 7b) clearly demonstrate that the pseudo-first-order model is suitable for describing the sonocatalytic degradation of MG in the M-ZLT@Co_3_O_4_/ultrasonic system. The consistent decrease in k_app_ with increasing initial MG concentration, together with the relatively high R^2^ values, confirms that the initial pollutant concentration is a critical parameter governing the degradation kinetics. These findings further suggest that lower MG concentrations favor faster sonocatalytic oxidation due to more efficient utilization of catalyst active sites and reactive species [83].

#### 2.2.3. Effect of pH

**Figure 8:**
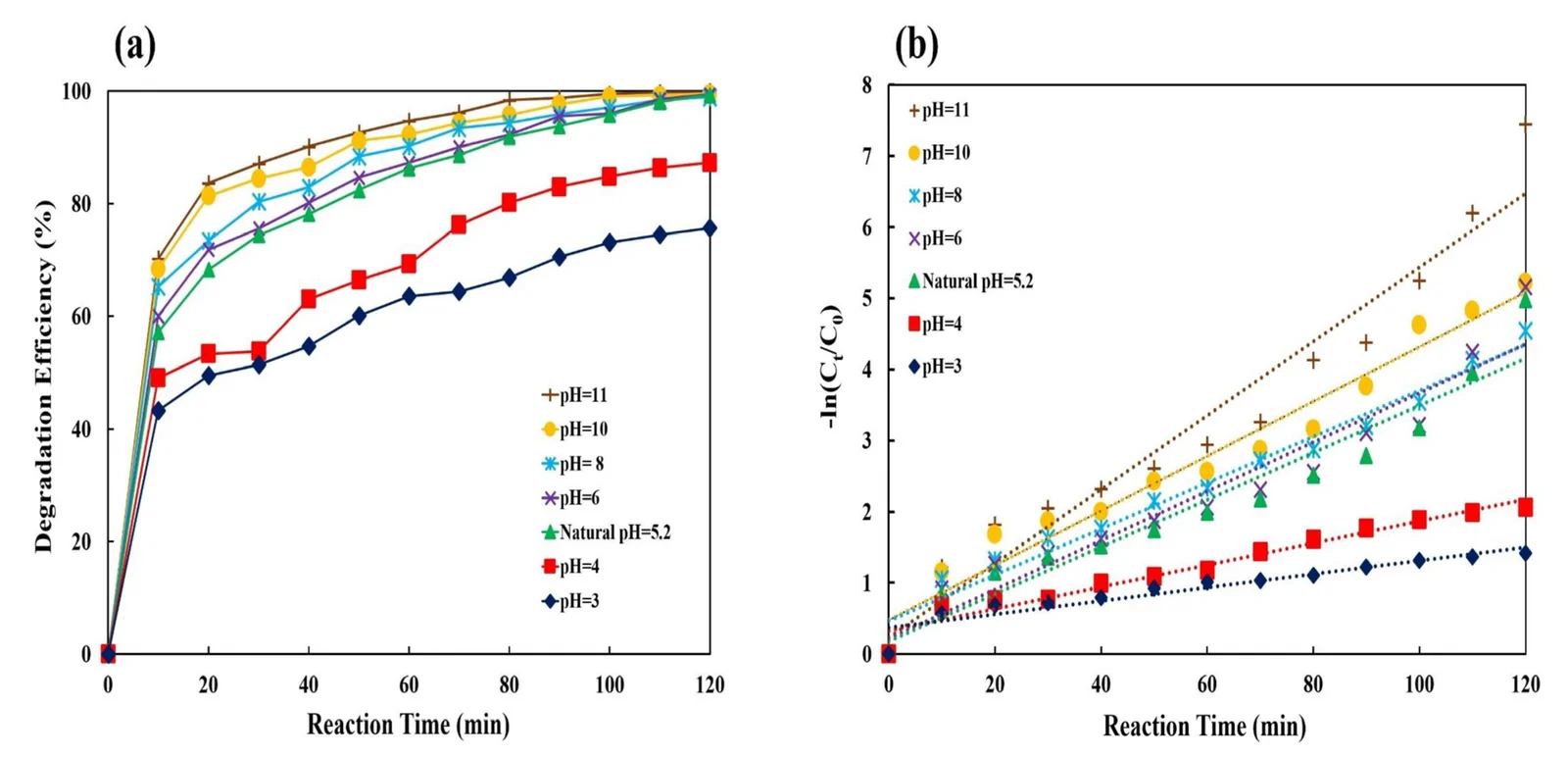
(**a**) The effect of pH on the degradation of MG the sonocatalytic degradation process, (**b**) Apparent pseudo-first-order reaction kinetics (Experimental Conditions: Initial MG concentration: 20 mg L^−1^, Catalyst dosage: 1.0 g L^−1^, Ultrasonic power: 300 W L^−1^, Reaction time: 120 min).

The initial solution pH is one of the most critical operational parameters in sonocatalytic degradation processes, since it directly affects the surface charge of the catalyst, the ionization state of the pollutant, adsorption affinity, cavitation behavior, and the formation of ROS. In this study, the effect of pH on the sonocatalytic degradation of MG was investigated in the presence of the M-ZLT@Co_3_O_4_ nanocatalyst under fixed experimental conditions, initial MG concentration of 20 mg L^−1^, catalyst dosage of 1.0 g L^−1^, ultrasonic power of 300 W L^−1^, and reaction time of 120 min. As shown in Figure 8a, the degradation efficiency increased markedly from 75.69% at pH 3 to 87.31% at pH 4, and then reached very high removal efficiencies at natural pH 5.2 and above. Specifically, the degradation efficiencies were 99.31%, 99.42%, 98.93%, 99.45%, and 99.94% at natural pH 5.2, pH 6, pH 8, pH 10, and pH 11, respectively. These results clearly indicate that acidic conditions negatively affect the sonocatalytic degradation of MG, whereas neutral to alkaline conditions strongly favor the degradation process [84].

The point of zero charge value of the M-ZLT@Co_3_O_4_ nanocatalyst was determined as pHpzc = 5.4 (Figure 14b). This value provides important insight into the interaction between MG molecules and the catalyst surface. At solution pH values lower than pHpzc, the catalyst surface is expected to be positively charged due to protonation of surface hydroxyl groups. Since MG is a cationic triphenylmethane dye, electrostatic repulsion between positively charged MG molecules and the positively charged catalyst surface may limit adsorption onto active sites under acidic conditions. In addition, excess H^+^ ions in acidic media can compete with MG molecules for adsorption sites and may also suppress the generation and stability of hydroxyl radicals [85]. These factors explain the relatively lower degradation efficiencies observed at pH 3 and pH 4. At pH values higher than pHpzc, the surface of M-ZLT@Co_3_O_4_ becomes negatively charged due to deprotonation of surface hydroxyl groups. This negatively charged surface can promote stronger electrostatic attraction toward cationic MG molecules, thereby improving their adsorption near catalytically active Co_3_O_4_ sites. This closer contact between MG molecules and the catalyst surface facilitates radical-mediated oxidation reactions. Moreover, alkaline conditions provide a higher concentration of OH^−^ ions, which can act as precursors for the formation of hydroxyl radicals under ultrasonic irradiation. During sonication, acoustic cavitation generates localized hot spots with extremely high temperature and pressure, leading to the sonolysis of water and dissolved oxygen. In the presence of M-ZLT@Co_3_O_4_, these processes are further enhanced through interfacial electron transfer and catalytic activation, producing reactive species such as •OH, •O_2_^−^, and related oxidative radicals. Therefore, the improved degradation efficiency under neutral and alkaline conditions can be attributed to the combined effects of enhanced electrostatic attraction, improved adsorption of MG, and intensified reactive oxygen species generation [86].

Interestingly, the degradation efficiency reached 99.31% at natural pH 5.2, which is very close to the pHpzc value of 5.4. Although the catalyst surface is expected to be nearly neutral or slightly positively charged under this condition, the very high removal efficiency suggests that the degradation mechanism is not governed solely by electrostatic adsorption. Instead, the excellent performance at natural pH indicates that M-ZLT@Co_3_O_4_ possesses strong intrinsic sonocatalytic activity. The zeolitic clay support may contribute to the preconcentration of MG molecules through porous adsorption, ion-exchange properties, and surface interactions, while Co_3_O_4_ acts as the active semiconductor phase responsible for accelerating charge transfer and reactive radical generation [87]. Therefore, even without external pH adjustment, the catalyst can efficiently degrade MG under environmentally relevant conditions. The slight differences observed between pH 5.2 and pH 11 indicate that, once the system reaches near-neutral or alkaline conditions, MG degradation proceeds almost completely. The efficiency increased only slightly from 99.31% at natural pH to 99.94% at pH 11, showing that excessive alkalization is not essential for achieving high degradation performance (Figure 8a). From an application perspective, this is a highly important finding because operating the process at natural pH eliminates the need for chemical pH adjustment, reduces operational cost, minimizes secondary chemical consumption, and improves the environmental compatibility of the treatment system. As a result, the pH dependent degradation behavior demonstrates that strongly acidic conditions are unfavorable for MG sonocatalytic degradation over M-ZLT@Co_3_O_4_ due to electrostatic repulsion, competition by H^+^ ions, and reduced radical generation efficiency. In contrast, neutral and alkaline conditions provide a more favorable catalytic environment by enhancing MG–catalyst interaction and reactive oxygen species formation. The nearly complete degradation obtained at natural pH highlights the practical applicability of the M-ZLT@Co_3_O_4_ nanocatalyst for the treatment of MG contaminated wastewater without the need for additional pH regulation [88].

As shown in Figure 8b, according to the kinetic results, the k_app_ values increased progressively with increasing solution pH. The rate constant increased from 0.0117 min^−1^ at pH 3 to 0.0172 min^−1^ at pH 4, indicating that strongly acidic conditions were less favorable for MG degradation. Under natural pH conditions, the k_app_ value reached 0.0414 min^−1^, demonstrating a significant enhancement in the sonocatalytic reaction rate without external pH adjustment. A further increase in pH resulted in higher kinetic constants, with values of 0.0430, 0.0378, 0.0435, and 0.0620 min^−1^ at pH 6, 8, 10, and 11, respectively (Figure 8b). This trend confirms that neutral to alkaline conditions strongly promote the sonocatalytic degradation rate of MG in the presence of M-ZLT@Co_3_O_4_. The increase in k_app_ with pH can be mainly attributed to the surface charge behavior of the catalyst and the ionic nature of MG. Since the pHpzc value of M-ZLT@Co_3_O_4_ was determined as 5.4, the catalyst surface is positively charged at pH values below 5.4 and negatively charged at pH values above 5.4. Under acidic conditions, the positively charged catalyst surface may electrostatically repel cationic MG molecules, reducing their adsorption on active catalytic sites. Moreover, excess H^+^ ions may compete with MG molecules for surface-active sites and suppress the generation of hydroxyl radicals. These effects explain the lower kapp values observed at pH 3 and pH 4. In contrast, when the solution pH exceeds the pHpzc, the M-ZLT@Co_3_O_4_ surface becomes negatively charged, favoring electrostatic attraction toward cationic MG molecules [89]. This enhanced interaction increases the local concentration of MG near the catalyst surface, thereby facilitating radical-mediated oxidation reactions. In addition, alkaline conditions provide more OH^−^ ions, which can promote the formation of highly oxidative hydroxyl radicals under ultrasonic irradiation. Therefore, the higher kapp values obtained at pH 8–11 indicate that alkaline conditions accelerate MG degradation by improving both adsorption affinity and reactive oxygen species generation [90].

The highest kapp value of 0.0620 min^−1^ was obtained at pH 11, indicating the fastest degradation rate under alkaline conditions. Compared with pH 3, the rate constant at pH 11 was approximately 5.29 times higher (Table 3c), clearly confirming the beneficial role of alkaline pH in enhancing the sonocatalytic performance of M-ZLT@Co_3_O_4_. However, the relatively high kapp value obtained at natural pH 5.2 also demonstrates that the catalyst exhibits strong intrinsic activity under near-neutral conditions, which is advantageous for practical wastewater treatment applications because it reduces the need for chemical pH adjustment [91]. The correlation coefficients (R^2^), ranging from 0.9307 to 0.9735, indicate that the pseudo-first-order kinetic model adequately describes the sonocatalytic degradation of MG under all investigated pH conditions. The highest R^2^ value was observed at pH 8 (R^2^ = 0.9735), suggesting a particularly good linear fit between −ln (C_t_/C_0_) and reaction time at this pH (Table 3c). Although some deviations from perfect linearity were observed, these may be attributed to the complexity of heterogeneous sonocatalytic systems, including simultaneous adsorption–desorption processes, mass-transfer effects, acoustic cavitation fluctuations, radical recombination, and the formation of intermediate degradation products [92].

As a result, the kinetic analysis confirms that the sonocatalytic degradation of MG over M-ZLT@Co_3_O_4_ follows pseudo-first-order behavior, and the reaction rate is strongly influenced by solution pH. The progressive increase in k_app_ from acidic to alkaline conditions demonstrates that higher pH values enhance the catalytic oxidation rate by promoting favorable electrostatic interactions between MG and the catalyst surface and by increasing reactive radical formation. These findings support the high sonocatalytic efficiency of M-ZLT@Co_3_O_4_, particularly under natural and alkaline pH conditions [93].

#### 2.2.4. Effect of Different Process on MG Degradation

**Figure 9:**
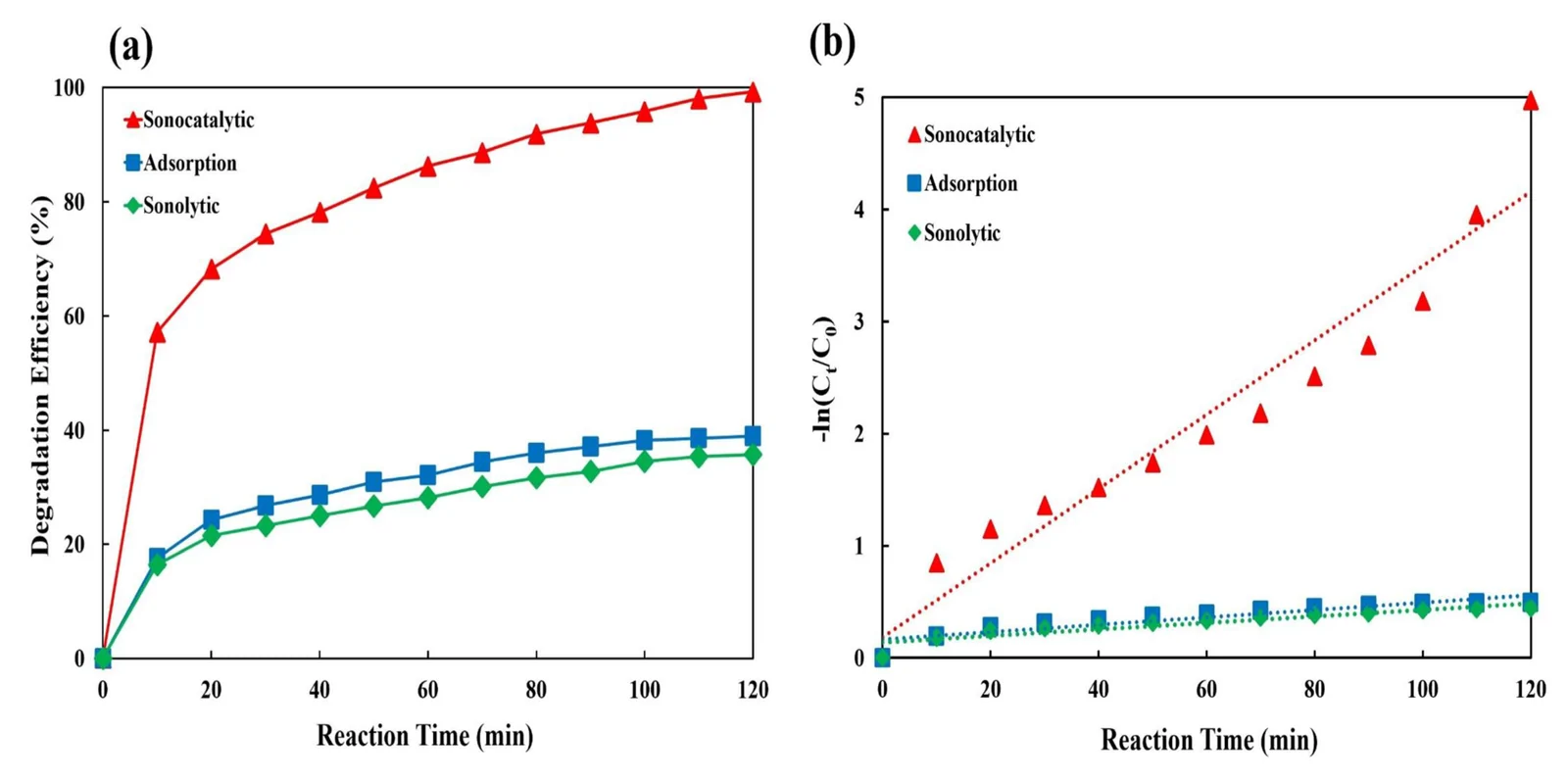
(**a**) Degradation effect of MG by sonocatalytic degradation process of different processes, (**b**) Apparent pseudo-first-order reaction kinetics (Experimental Conditions: Initial MG concentration: 20 mg L^−1^, catalyst dosage: 1.0 g L^−1^, pH for MG: Natural pH, Ultrasonic power: 300 W L^−1^, Reaction time: 120 min).

As shown in Figure 9a, the highest degradation efficiency was obtained in the sonocatalytic process using the M-ZLT@Co_3_O_4_ nanocatalyst, reaching 99.31% MG removal after 120 min. In contrast, adsorption alone resulted in only 38.91% removal, while the sonolytic process without catalyst achieved 35.71% degradation. These results clearly demonstrate that neither adsorption nor ultrasound irradiation alone is sufficient to achieve effective MG elimination under the same operating conditions. The relatively low degradation efficiency observed in the adsorption process indicates that the contribution of surface adsorption to the overall MG removal is limited [94]. Although the porous structure and active surface sites of M-ZLT@Co_3_O_4_ can adsorb MG molecules to some extent, adsorption alone cannot provide sufficient degradation or mineralization of the dye molecules. Similarly, the limited degradation efficiency obtained during sonolysis can be attributed to the insufficient generation of reactive oxygen species solely by ultrasonic cavitation. In the absence of a catalyst, ultrasound-induced cavitation produces hydroxyl radicals and localized hot spots; however, the amount of reactive species generated is not adequate to achieve complete MG degradation within 120 min [95]. The remarkable enhancement observed in the sonocatalytic process confirms the strong synergistic interaction between ultrasonic irradiation and the M-ZLT@Co_3_O_4_ hybrid nanocatalyst. Under ultrasonic irradiation, cavitation bubbles collapse violently, generating high-temperature and high-pressure micro environments that promote the formation of reactive oxygen species such as •OH and •O_2_^−^ radicals. The presence of M-ZLT@Co_3_O_4_ further enhances this process by providing additional active sites, improving the contact between MG molecules and reactive species, and facilitating charge separation on the Co_3_O_4_ surface [96]. Moreover, the zeolite supported structure may contribute to better dispersion of Co_3_O_4_ nanoparticles, increased surface accessibility, and improved catalytic stability, thereby promoting efficient MG degradation. Therefore, the much higher degradation efficiency achieved by the M-ZLT@Co_3_O_4_-assisted sonocatalytic system compared with adsorption and sonolysis confirms that MG degradation mainly proceeds through a catalytic oxidation pathway rather than simple physical adsorption or direct ultrasound-induced decomposition. These findings highlight the superior catalytic role of M-ZLT@Co_3_O_4_ and demonstrate its potential as an efficient sonocatalyst for the treatment of dye-contaminated wastewater [97,98].

As shown in Figure 9b, the sonocatalytic process exhibited the highest kapp value of 0.0847 min^−1^ with an R^2^ value of 0.9325. This result confirms the markedly faster degradation of MG in the presence of the M-ZLT@Co_3_O_4_ nanocatalyst under ultrasonic irradiation. In comparison, the adsorption and sonolytic processes showed much lower k_app_ values of 0.0041 and 0.0036 min^−1^, respectively (Table 3d). The apparent rate constant of the sonocatalytic system was approximately 10.09 times higher than that of adsorption and 11.5 times higher than that of sonolysis [99]. This substantial enhancement indicates that the removal of MG is not governed only by surface adsorption or direct ultrasonic decomposition, but mainly by the catalytic activation of the M-ZLT@Co_3_O_4_ surface under ultrasonic irradiation. Although adsorption and sonolysis exhibited relatively high R^2^ values of 0.9589 and 0.9761, respectively, their low k_app_ values demonstrate that these processes proceeded at much slower rates (Table 3d) [100]. Therefore, higher linearity does not necessarily indicate better degradation performance; rather, it only shows that the concentration decay followed pseudo-first-order behavior. In contrast, the slightly lower R^2^ value observed for the sonocatalytic process may be associated with the more complex degradation pathway involving adsorption, cavitation effects, radical generation, and catalytic oxidation occurring simultaneously [101]. The calculated kinetic parameters are consistent with the degradation efficiency results, where the M-ZLT@Co_3_O_4_-assisted sonocatalytic system achieved almost complete MG degradation within 120 min, while adsorption and sonolysis alone were significantly less effective (Figure 9b). These findings clearly demonstrate the strong synergistic effect between ultrasonic irradiation and the M-ZLT@Co_3_O_4_ nanocatalyst. Therefore, the high kapp value and acceptable pseudo-first-order fitting confirm that M-ZLT@Co_3_O_4_ is an efficient sonocatalyst for the rapid degradation of MG from aqueous media [102].

#### 2.2.5. Effect of Water Matrix on the Sonocatalytic Degradation of MG

**Figure 10:**
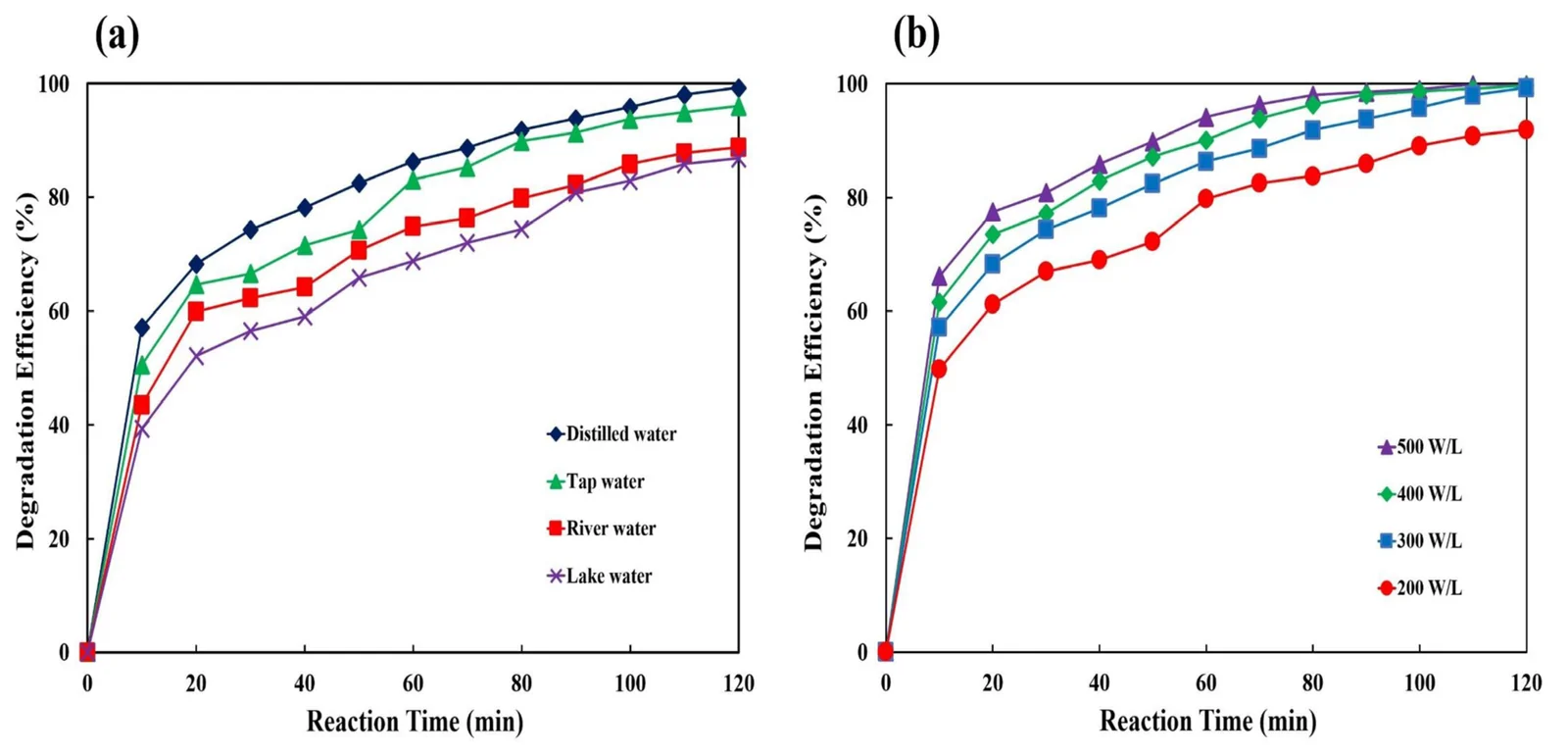
(**a**) Effect of water matrix, (**b**) Effect of ultrasonic power on the sonocatalytic degradation of MG in the presence of M-ZLT@Co_3_O_4_ hybrid nanocatalyst (Experimental Conditions: Initial MG concentration: 20 mg L^−1^, Catalyst amount: 1.0 g L^−1^, pH for MG: Natural pH, Ultrasonic power: 300 W L^−1^, Reaction time: 120 min).

The influence of the water matrix on the sonocatalytic degradation of Malachite Green (MG) was systematically evaluated using the M-ZLT@Co_3_O_4_ nanocatalyst under the following experimental conditions, initial MG concentration of 20 mg L^−1^, catalyst dosage of 1.0 g L^−1^, natural pH, ultrasonic power of 300 W L^−1^, and reaction time of 120 min. As shown in Figure 10a, the degradation efficiencies obtained in different aqueous media followed the order, distilled water (99.31%) > tap water (96.08%) > river water (88.84%) > lake water (86.90%). These findings clearly demonstrate that the composition of the water matrix plays a decisive role in governing the sonocatalytic performance of the catalyst [103].

The physicochemical characteristics of the real water matrices employed in the sonocatalytic experiments, including relevant parameters such as pH, electrical conductivity, total dissolved solids, TOC and major ionic constituents, are summarized in Table 4.

The almost complete degradation observed in distilled water indicates that, in the absence of interfering inorganic ions, dissolved organic matter, and suspended particulates, the generated reactive oxygen species (ROS) can efficiently attack the MG molecules. Under ultrasonic irradiation, acoustic cavitation produces localized hot spots with extremely high transient temperatures and pressures, promoting the homolytic cleavage of water molecules and the formation of highly oxidative radicals. The generated hydroxyl radicals (•OH) and superoxide radicals (•O_2_^−^), are the major oxidizing species responsible for MG degradation [104]. The overall degradation process can therefore be summarized as follows in Equation (15).

*MG + •OH/•O*_2_^−^ → *Intermediate products → CO*_2_ + *H*_2_*O + Inorganic ions* (15)

The high activity of M-ZLT@Co_3_O_4_ can be attributed to the synergistic combination of the Co_3_O_4_ active phase and the modified zeolite support. Co_3_O_4_ provides catalytically active sites for ROS generation, while the M-ZLT support enhances surface dispersion of Co_3_O_4_ particles, facilitates adsorption of MG molecules, and improves mass transfer at the solid–liquid interface. Moreover, ultrasonic cavitation can continuously clean and reactivate the catalyst surface, thereby sustaining catalytic performance. In tap water, the degradation efficiency decreased only slightly to 96.08%, suggesting that the nanocatalyst retains high activity even in the presence of moderate levels of dissolved ions [105]. Nevertheless, the small reduction compared with distilled water may be attributed to the scavenging action of common ions such as bicarbonate, carbonate, and chloride, which can consume hydroxyl radicals and reduce their availability for MG oxidation. Representative radical scavenger reactions include as follows in Equations (16)–(18).

*HCO*_3_^−^ + *•OH → CO*_3_•^−^ + *H*_2_*O* (16)

*CO*_3_^2−^ + *•OH → CO*_3_•^−^ + *OH^−^* (17)

*Cl^−^ + •OH → ClOH•^−^* (18)

These side reactions lower the effective ROS concentration in solution and consequently suppress the degradation rate to some extent. A more pronounced decline in degradation efficiency was observed in river water (88.84%) and lake water (86.90%) (Figure 10a). This decrease is most likely associated with the greater chemical complexity of natural waters, which typically contain higher concentrations of natural organic matter (NOM), dissolved salts, colloidal particles, and suspended solids. These constituents can adversely affect sonocatalytic degradation through several mechanisms. First, NOM competes with MG for the reactive radicals as follows in Equation (19) [106].

*NOM + •OH → Oxidized NOM products* (19)

Second, inorganic anions act as radical scavengers, as shown above. Third, suspended particles and colloids may hinder mass transfer, partially block catalyst active sites, or attenuate the propagation of ultrasonic waves, thereby weakening cavitation intensity. Fourth, naturally occurring cations and organic substances may alter the surface charge characteristics of the catalyst, influencing dye adsorption and interfacial oxidation processes. The slightly lower efficiency obtained in lake water than in river water may indicate a stronger inhibitory effect of dissolved organic matter and stagnant water associated contaminants in the lake matrix. However, unless the exact physicochemical composition of these waters is analytically determined, this interpretation should be regarded as a mechanistically reasonable explanation rather than a definitive conclusion [3]. From an application perspective, these results are highly significant because they demonstrate that M-ZLT@Co_3_O_4_ remains remarkably effective in real and semi-real water environments, despite the expected matrix-related suppression effects. Achieving more than 86% MG degradation in lake water and nearly 89% in river water under non-optimized natural matrix conditions confirms the strong practical potential of this nanocatalyst for the treatment of dye-contaminated environmental waters. At the same time, the superior performance in distilled water confirms that the intrinsic sonocatalytic activity of the catalyst is very high, while the differences among the matrices mainly arise from external water chemistry effects rather than catalyst instability [107].

Result of the water matrix study confirms that the sonocatalytic degradation of MG by M-ZLT@Co_3_O_4_ nanocatalyst is highly efficient but sensitive to the presence of radical scavengers and competing constituents in natural waters. The observed efficiency order, distilled water > tap water > river water > lake water, is consistent with the progressive increase in matrix complexity (Figure 10a). These findings further support that ROS-driven oxidation is the dominant degradation pathway and that optimization strategies for real wastewater treatment should focus on mitigating matrix interference, improving ROS utilization, and preserving catalyst surface accessibility [108].

#### 2.2.6. Effect of Ultrasonic Power on the Sonocatalytic Degradation of MG

As shown in Figure 10b, the effect of ultrasonic power on the sonocatalytic degradation of MG was investigated in the presence of the M-ZLT@Co_3_O_4_ nanocatalyst under fixed experimental conditions, and the obtained degradation efficiencies after 120 min followed the order, 500 W L^−1^ (99.90%) > 400 W L^−1^ (99.74%) > 300 W L^−1^ (99.31%) > 200 W L^−1^ (91.98%). These results clearly demonstrate that ultrasonic power is a highly influential operational parameter in the sonocatalytic process, as it directly governs cavitation intensity, reactive oxygen species generation, mass-transfer behavior, and catalyst surface activation [93].

At the lowest applied power, 200 W L^−1^, the degradation efficiency remained at 91.98%, which already indicates a substantial sonocatalytic activity of the M-ZLT@Co_3_O_4_ system. However, this lower efficiency compared with the other power levels suggests that the cavitation phenomenon was less intense under these conditions. At insufficient ultrasonic power, fewer cavitation bubbles are generated, and their collapse is less violent, resulting in lower localized temperature–pressure extremes and consequently lower radical production. In addition, weaker acoustic streaming and microturbulence may limit the transport of MG molecules from the bulk solution to the catalyst surface, thereby reducing the overall degradation performance [109]. When the ultrasonic power was increased from 200 to 300 W L^−1^, the degradation efficiency rose markedly from 91.98% to 99.31%, indicating a substantial intensification of the sonocatalytic process (Figure 10b). This strong increase suggests that 300 W L^−1^ provides sufficient acoustic energy to induce more effective cavitation, enhance the frequency of bubble implosion events, and promote stronger interfacial interactions between MG molecules and the active sites of the nanocatalyst. Moreover, at higher ultrasonic power, the shock waves and microjets generated during bubble collapse may contribute to continuous cleaning and reactivation of the catalyst surface by removing adsorbed by-products and preventing passivation of active centers [110].

A further increase in ultrasonic power to 400 W L^−1^ and 500 W L^−1^ led to only slight improvements in degradation efficiency, reaching 99.74% and 99.90%, respectively (Figure 10b). This behavior indicates that, although higher power strengthens cavitation and radical generation, the system approaches a near-saturation region above 300 W L^−1^. In this process, most MG molecules can already be efficiently attacked and decomposed, and therefore additional acoustic energy produces only marginal gains in overall degradation efficiency. Such a trend is commonly observed in advanced sonochemical and sonocatalytic systems, where the relationship between ultrasonic power and degradation performance becomes non-linear at elevated power densities [111]. The slight difference between 300, 400, and 500 W L^−1^ is particularly important from a mechanistic and practical perspective. It suggests that the M-ZLT@Co_3_O_4_ nanocatalyst possesses a highly efficient catalytic structure capable of utilizing sonochemically generated reactive species effectively even at moderate ultrasonic input. The Co_3_O_4_ component likely serves as the main active phase for radical-mediated oxidation, while the modified zeolite support (M-ZLT) contributes to enhanced dye adsorption, better dispersion of Co_3_O_4_ nanoparticles, and improved accessibility of active sites. Under ultrasound irradiation, this synergistic structure facilitates rapid oxidation of MG, allowing the system to achieve near-complete degradation even before the highest power level is reached [112].

Another important point is that increasing ultrasonic power not only increases ROS formation but also improves physical transport phenomena in the reaction medium. Stronger acoustic waves generate more vigorous mixing, thinner diffusion boundary layers, and improved contact between pollutant molecules and catalyst particles. In heterogeneous sonocatalytic systems, this is especially important because degradation is controlled not only by radical generation but also by how efficiently the pollutant reaches the catalyst surface and interacts with the reactive sites. Thus, the improvement observed with increasing power arises from the combined chemical and physical effects of ultrasound [113]. However, although 500 W L^−1^ provided the highest degradation efficiency, the difference relative to 400 W L^−1^ and 300 W L^−1^ was very small. Specifically, the increase from 300 to 500 W L^−1^ resulted in only a 0.59% improvement, which indicates that the process had already become highly efficient at 300 W L^−1^. Therefore, from an energy-efficiency standpoint, one may infer that 300 W L^−1^ or 400 W L^−1^ could be more practically favorable operating powers since they deliver almost complete degradation while potentially requiring lower energy consumption than 500 W L^−1^. This interpretation is particularly relevant for large-scale applications, where process economics and energy demand are critical considerations. Nevertheless, if the objective is to achieve the absolute maximum removal efficiency regardless of energy cost, 500 W L^−1^ can be considered the most effective condition among the tested values [114].

Overall, the results confirm that ultrasonic power plays a central role in controlling the sonocatalytic degradation of MG by M-ZLT@Co_3_O_4_, mainly through its influence on cavitation intensity, reactive species production, catalyst surface activation, and mass-transfer enhancement. The observed efficiency sequence, 500 W L^−1^ > 400 W L^−1^ > 300 W L^−1^ > 200 W L^−1^ (Figure 10b), is fully consistent with the expected behavior of ultrasound-assisted advanced oxidation systems. At lower power, degradation is limited by insufficient cavitation activity, whereas at higher power the system reaches a nearly complete degradation due to abundant ROS generation and improved catalyst–pollutant interactions. These findings demonstrate the strong responsiveness of the M-ZLT@Co_3_O_4_ nanocatalyst to ultrasonic irradiation and further confirm its high potential as an efficient sonocatalyst for the treatment of dye-contaminated wastewater [115].

#### 2.2.7. Effect of Inorganic Radical Scavengers

**Figure 11:**
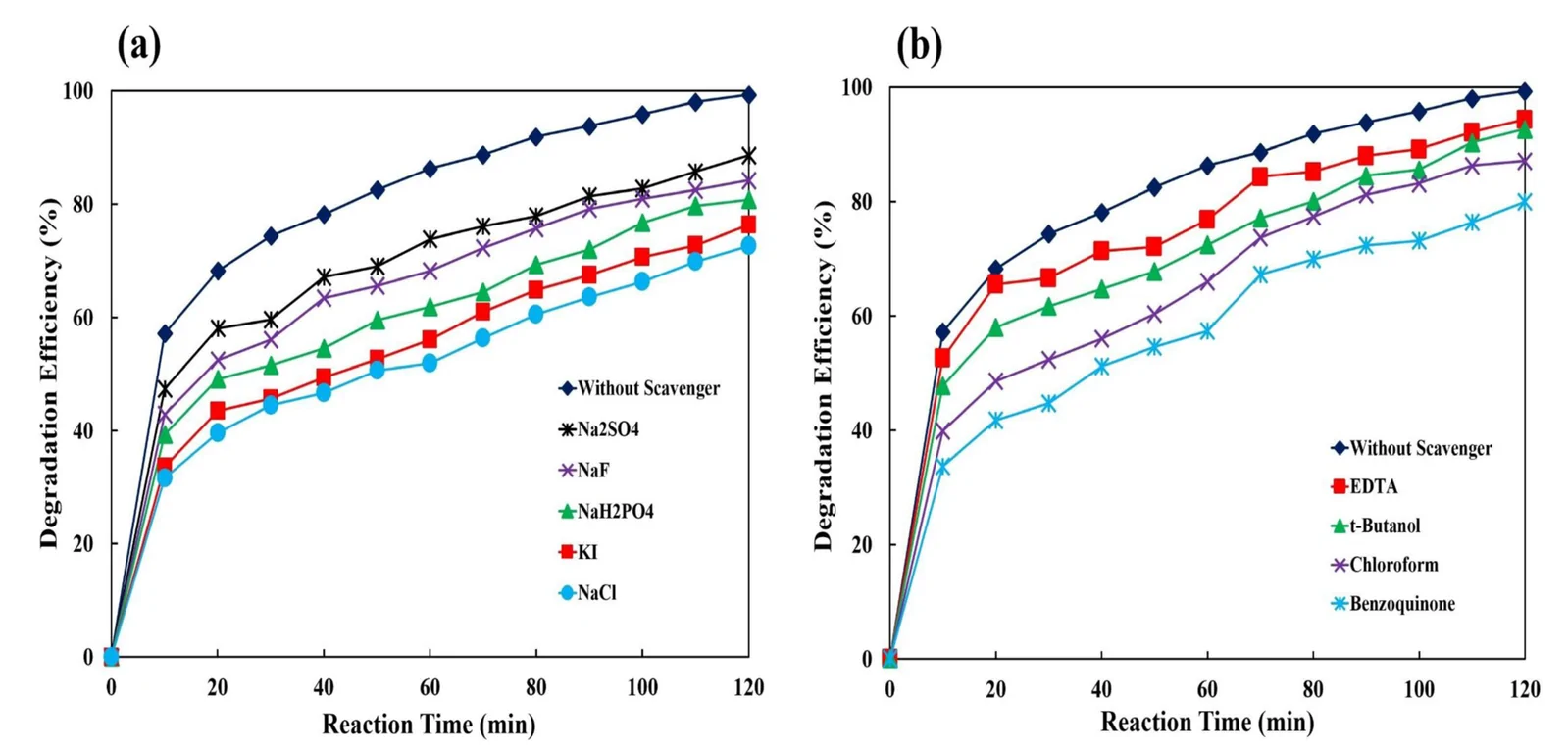
(**a**) Effect of inorganic radical scavengers on the degradation of MG by sonocatalytic degradation process, (**b**) Effect of organic radical scavengers on the degradation of MG by sonocatalytic degradation process (Experimental conditions: Initial MG concentration: 20 mg L^−1^, Catalyst dosage: 1.0 g L^−1^, pH for MG: Natural pH, Radical scavenger concentration: 20 mg L^−1^, Ultrasonic power: 300 W L^−1^, Reaction time: 120 min).

As shown in Figure 11a, under the optimized sonocatalytic conditions, the M-ZLT@Co_3_O_4_ nanocatalyst exhibited a very high MG degradation efficiency of 99.31% after 120 min in the absence of any scavenger. However, the addition of inorganic scavengers caused a clear decrease in degradation performance, confirming that the degradation of Malachite Green is mainly controlled by reactive oxygen species and surface-mediated oxidation pathways. The inhibition effect followed the order, NaCl > KI > NaH_2_PO_4_ > NaF > Na_2_SO_4_ with degradation efficiencies decreasing to 72.65%, 76.31%, 80.72%, 84.20%, and 88.60%, respectively (Figure 11a). This trend indicates that hydroxyl radicals (•OH), surface holes (h^+^), and secondary radical species generated during ultrasonic cavitation play decisive roles in MG oxidation. The addition of Na_2_SO_4_ decreased MG degradation from 99.31% to 88.60%, showing the weakest inhibition among the tested inorganic scavengers. Sulfate ions can react with hydroxyl radicals and surface holes as follows Equations (20) and (21) [116].

*SO*_4_^2−^ + •*OH → SO*_4_•^−^ + *OH^−^* (20)

*SO*_4_^2−^ + *h^+^ → SO*_4_•^−^ (21)

Although sulfate consumes part of the generated •OH and h^+^, the resulting sulfate radical (SO_4_•^−^) is still a relatively strong oxidizing species. Therefore, Na_2_SO_4_ does not fully suppress MG degradation. The moderate decrease suggests that sulfate ions partially compete with MG for reactive sites but do not completely terminate the oxidative pathway. In the presence of NaF, the degradation efficiency decreased to 84.20%. Fluoride ions are commonly associated with the suppression of surface-bound hydroxyl radicals (•OH) and surface holes because they can interact strongly with metal oxide surface hydroxyl groups [117]. By replacing surface –OH groups or blocking active sites on Co_3_O_4_, fluoride can reduce the generation of surface-bound radicals. Therefore, the decrease observed in the presence of NaF indicates that surface hydroxyl radicals generated at the M-ZLT@Co_3_O_4_ interface significantly contribute to MG degradation. The addition of NaH_2_PO_4_ reduced the degradation efficiency to 80.72%, indicating stronger inhibition than sulfate and fluoride. Dihydrogen phosphate ions can consume hydroxyl radicals and holes through as follows Equations (22) and (23) [118].

*H*_2_*PO*_4_^−^ + *•OH → HPO*_4_•^−^ + *H*_2_O (22)

*H*_2_*PO*_4_^−^ + *h*^+^ → *H*_2_*PO*_4_• (23)

Phosphate species may also adsorb onto positively charged or hydroxylated catalyst surfaces, thereby blocking active catalytic sites. This reduces MG adsorption near the catalyst surface and limits the interaction between MG molecules and ROS. Hence, the relatively strong inhibition by NaH_2_PO_4_ confirms that •OH radicals and surface-active sites are essential for efficient MG oxidation [119]. The presence of KI decreased MG degradation to 76.31%, showing a pronounced scavenging effect. Iodide ions are efficient scavengers for both hydroxyl radicals and sonogenerated holes as follows in Equations (24)–(27).

*I^−^ + •OH → I• + OH^−^* (24)

*I^−^ + h^+^ →I•* (25)

*I• + I^−^ → I*_2_•^−^ (26)

*I*_2_•^−^ + *I*_2_
*→ I*_3_^−^ (27)

The strong decrease in degradation efficiency in the presence of KI indicates that holes and hydroxyl radicals are strongly involved in the sonocatalytic degradation of MG. Since iodide rapidly captures oxidative species, fewer ROS remain available to attack MG molecules, leading to a significant decrease in degradation performance. NaCl caused the strongest inhibition, reducing MG degradation to 72.65%. Chloride ions can rapidly react with hydroxyl radicals and holes to form chlorine-based radical intermediates as follows in Equations (28)–(31) [120].

*Cl^−^ + •OH → ClOH•^−^* (28)

*ClOH•^−^ → Cl• + OH^−^* (29)

*Cl• + Cl^−^ → Cl*_2_•^−^ (30)

*Cl^−^ + h^+^ →Cl•* (31)

Although chlorine radicals such as Cl• and Cl_2_•^−^ can exhibit some oxidative ability, they are generally less effective and less non-selective than hydroxyl radicals for the rapid destruction of complex dye molecules. In addition, chloride ions may compete with MG molecules for active catalytic sites and reduce the availability of highly reactive •OH radicals. Therefore, the strong inhibition observed with NaCl demonstrates that •OH mediated oxidation is the dominant pathway in MG sonocatalytic degradation over M-ZLT@Co_3_O_4_. The scavenger results clearly demonstrate that MG degradation over M-ZLT@Co_3_O_4_ is governed by a radical-driven sonocatalytic oxidation mechanism [121]. The strong suppression caused by NaCl and KI confirms the important contribution of •OH radicals and holes, while the inhibition observed with NaH_2_PO_4_ and NaF further supports the participation of surface-bound hydroxyl radicals and catalyst active sites. The relatively weaker effect of Na_2_SO_4_ suggests that sulfate ions partially consume oxidative species but may generate secondary sulfate radicals that still contribute to oxidation [122].

These results indicate that the high sonocatalytic activity of M-ZLT@Co_3_O_4_ originates from the synergistic interaction between ultrasonic cavitation, Co_3_O_4_ redox cycling, surface hydroxyl activation, and the adsorption/dispersion capability of the CTAB-modified zeolite clay support. The inorganic scavenger experiments provide strong mechanistic evidence that hydroxyl radicals are the principal oxidative species, while holes and secondary oxygen-centered radicals also participate in the degradation of MG [123].

#### 2.2.8. Effect of Organic Radical Scavengers

As shown in Figure 11b, the influence of organic radical scavengers was investigated to identify the dominant reactive species involved in the sonocatalytic degradation of (MG) over the M-ZLT@Co_3_O_4_ nanocatalyst. Under scavenger free conditions, the degradation efficiency reached 99.31% after 120 min, confirming the excellent sonocatalytic activity of M-ZLT@Co_3_O_4_. However, the addition of organic scavengers reduced the degradation efficiency to different extents, indicating that several ROS and charge carriers participate in the degradation process. The inhibition effect followed the order, Benzoquinone > Chloroform > t-Butanol > EDTA with degradation efficiencies of 79.97%, 87.16%, 92.68%, and 94.44%, respectively (Figure 11b). This trend suggests that superoxide radicals (•O_2_^−^) play the most significant role, while electrons (e^−^), hydroxyl radicals (•OH), and holes (h^+^) also contribute to the degradation of MG [124].

EDTA is commonly used as a hole scavenger. In the presence of EDTA, the degradation efficiency decreased only slightly from 99.31% to 94.44%. This limited inhibition indicates that holes contribute to the degradation process, but they are not the dominant oxidative species. By consuming surface generated holes, EDTA reduces the direct oxidation of MG and also suppresses the formation of hydroxyl radicals from surface hydroxyl groups. The hole-scavenging reaction can be expressed as follows in Equations (32) and (33) [125].

*EDTA + h^+^ → EDTA•^+^* (32)

*H*_2_*O/OH*^−^ + *h*^+^ → •*OH* (33)

However, because the inhibition was relatively weak, the degradation of MG over M-ZLT@Co_3_O_4_ is not mainly controlled by direct hole oxidation. Instead, holes may act as secondary contributors by assisting •OH generation at the catalyst surface. t-Butanol is a well-known hydroxyl radical scavenger. The addition of t-butanol decreased the MG degradation efficiency to 92.68%, confirming the participation of •OH radicals in the sonocatalytic oxidation process. The scavenging reaction is as follows in Equation (34) [126].

*(CH*_3_*)*_3_*COH + •OH → (CH*_3_*)*_2_*C•OHCH*_3_ + *H*_2_*O* (34)

Hydroxyl radicals are highly reactive and non-selective oxidizing species that can attack the chromophoric structure of MG, leading to cleavage of the conjugated system, N-demethylation, aromatic ring opening, and formation of smaller degradation intermediates. The moderate inhibition caused by t-butanol indicates that •OH radicals are important but not the only dominant species in the M-ZLT@Co_3_O_4_/US system. Chloroform is generally considered an electron scavenger and can suppress electron mediated oxygen activation [127]. In the presence of chloroform, MG degradation decreased to 87.16%, showing a stronger inhibitory effect than EDTA and t-butanol. The electron scavenging reaction may be represented as follows in Equations (35) and (36).

*CHCl*_3_ *+ e^−^ → CHCl*_3_•^−^ (35)

*CHCl*_3_•^−^ *→ •CHCl*_2_ *+ Cl^−^* (36)

Benzoquinone caused the strongest inhibition among the tested organic scavengers, decreasing the MG degradation efficiency from 99.31% to 79.97%. Benzoquinone is widely used as a superoxide radical scavenger. Therefore, this pronounced decrease strongly suggests that •O_2_^−^ radicals are the principal reactive species in the sonocatalytic degradation of MG over M-ZLT@Co_3_O_4_. The scavenging reaction can be expressed as follows in Equation (37) [128].

*BQ + •O*_2_^−^ *→ BQ•^−^ + O*_2_ (37)

The strong inhibition by benzoquinone indicates that superoxide radicals generated through oxygen reduction are highly involved in MG degradation. These radicals can directly oxidize MG or participate in further ROS formation through protonation pathway as follows Equations (38)–(40).

*•O*_2_^−^ *+ H^+^ → HO*_2_• (38)

2*HO*_2_*• → H*_2_*O*_2_ + *O*_2_ (39)

*H*_2_*O*_2_ + *e^−^ → •OH + OH^−^* (40)

Thus, suppression of •O_2_^−^ formation also limits the generation of secondary oxidative species such as HO_2_•, H_2_O_2_, and •OH, explaining the significant decrease in degradation efficiency. The dominant role of superoxide radicals (•O_2_^−^) suggests that the oxygen activation pathway is highly important in the M-ZLT@Co_3_O_4_-assisted sonocatalytic system. Under ultrasonic irradiation, electron–hole pairs and cavitation induced radicals are generated. Electrons react with dissolved oxygen to produce •O_2_^−^, while holes and water oxidation produce •OH radicals [129]. The Co_3_O_4_ phase further promotes redox cycling through Co^2+^/Co^3+^ sites, enhancing ROS formation. Therefore, the scavenger study demonstrates that MG degradation by M-ZLT@Co_3_O_4_ is not controlled by a single oxidative species, but proceeds through a multi-radical sonocatalytic mechanism. Among these species, •O_2_^−^ radicals are the most influential, while •OH radicals and holes act as complementary oxidative species. The strong performance of M-ZLT@Co_3_O_4_ can be attributed to the synergistic effects of ultrasonic cavitation, Co_3_O_4_-mediated redox reactions, enhanced oxygen activation, and the adsorption capacity of the CTAB-modified zeolite support [130].

#### 2.2.9. Reusability of the M-ZLT@Co_3_O_4_ Nanocatalyst in the Sonocatalytic Degradation of MG

**Figure 12:**
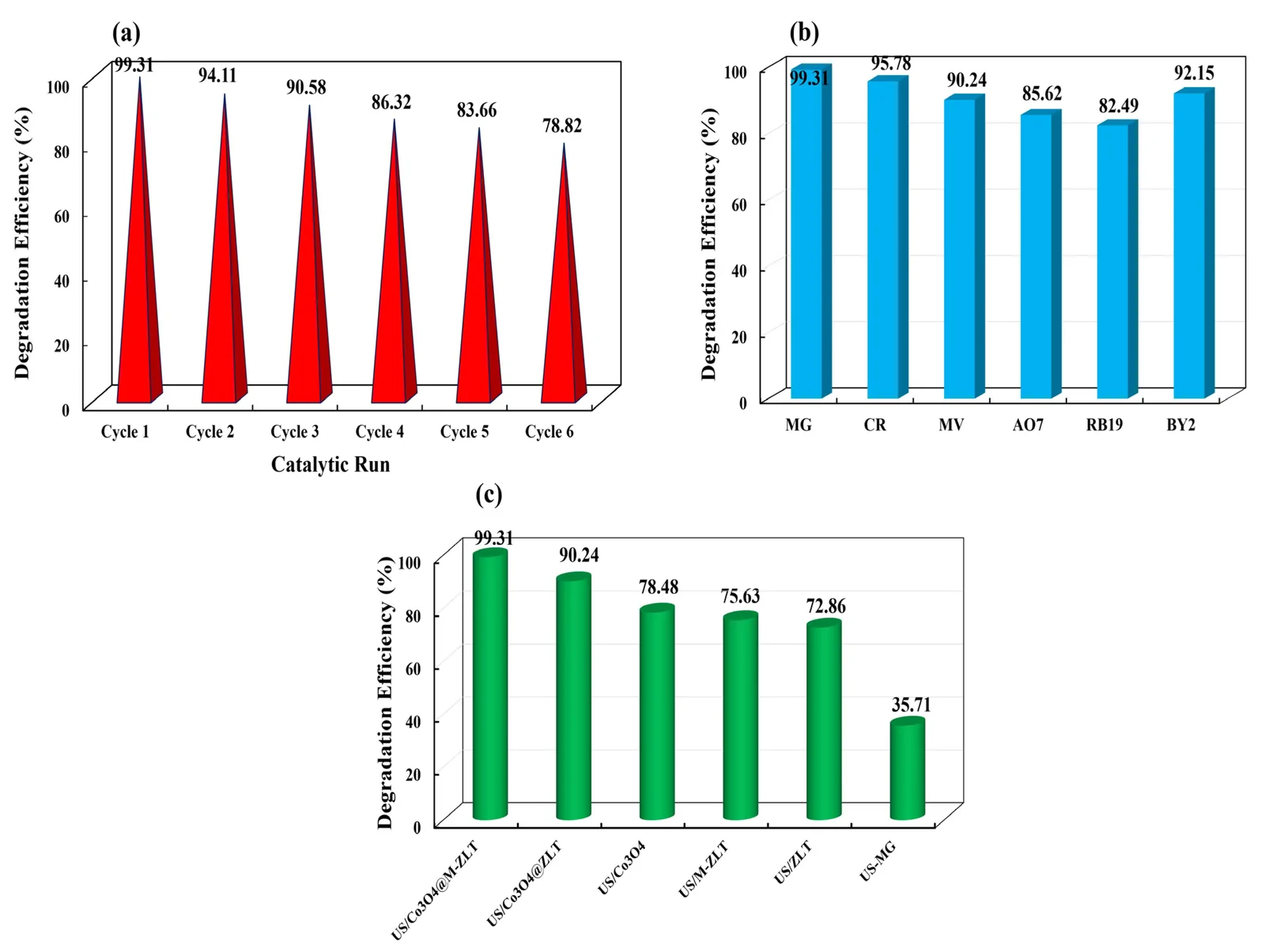
(**a**) Reusability of the M-ZLT@Co_3_O_4_ nanocatalyst by sonocatalytic degradation process, (**b**) Effect of sonocatalytic degradation different dyes in the presence of M-ZLT@Co_3_O_4_ nanocatalyst, (**c**) Degradation effect of MG by sonocatalytic degradation process of different catalysts (Experimental Conditions: Initial dye concentration: 20 mg L^−1^, Catalyst dosage: 1.0 g L^−1^, pH for MG: Natural pH, Ultrasonic power: 300 W L^−1^, Reaction time: 120 min).

As shown in Figure 12a, under the optimized sonocatalytic conditions, including an initial MG concentration of 20 mg L^−1^, M-ZLT@Co_3_O_4_ dosage of 1.0 g L^−1^, natural solution pH, ultrasonic power of 300 W L^−1^, and a reaction time of 120 min, the reusability performance of the M-ZLT@Co_3_O_4_ nanocatalyst was investigated over six consecutive degradation cycles. Reusability is a critical parameter for evaluating the practical applicability, operational stability, and economic feasibility of heterogeneous sonocatalysts in wastewater treatment processes [131]. The M-ZLT@Co_3_O_4_ nanocatalyst exhibited a very high MG degradation efficiency of 99.31% in the first cycle, confirming its excellent initial sonocatalytic activity. After repeated use, the degradation efficiency gradually decreased to 94.11%, 90.58%, 86.32%, 83.66%, and 78.82% from the second to the sixth cycle, respectively (Figure 12a). Although a progressive decline in activity was observed, the catalyst still maintained a considerable sonocatalytic degradation efficiency of nearly 79% after six consecutive cycles. This result indicates that the M-ZLT@Co_3_O_4_ nanocatalyst possesses acceptable reusability and maintains a significant portion of its catalytic activity under repeated sonochemical operation [132].

The decrease in degradation performance during successive cycles can be attributed to several possible factors. First, the repeated exposure of the catalyst surface to ultrasonic irradiation may cause partial surface erosion, structural fatigue, or detachment of active Co_3_O_4_ nanoparticles from the M-ZLT support. Second, the adsorption of MG molecules and degradation intermediates onto the catalyst surface may block active catalytic sites, thereby reducing the interaction between the pollutant molecules and reactive oxygen species. In addition, incomplete removal of residual organic species during the washing and recovery steps may lead to gradual surface fouling [133]. Minor catalyst loss during centrifugation, filtration, washing, and drying processes may also contribute to the observed reduction in sonocatalytic efficiency. From a mechanistic perspective, the high initial activity of M-ZLT@Co_3_O_4_ can be related to the synergistic interaction between Co_3_O_4_ nanoparticles and the CTAB-modified zeolite clay support. The M-ZLT matrix provides a suitable support structure, improves pollutant adsorption near the catalyst surface, and facilitates the dispersion of Co_3_O_4_ active sites. Under ultrasonic irradiation, acoustic cavitation generates localized high-temperature and high-pressure micro environments, promoting the formation of reactive oxygen species such as •OH and •O_2_^−^ radicals. These oxidative species play a key role in the degradation of MG molecules. However, after repeated cycles, partial blockage or deactivation of these active sites may reduce ROS generation efficiency, leading to lower degradation performance [134].

The retention of 78.82% degradation efficiency after the sixth cycle demonstrates that the M-ZLT@Co_3_O_4_ nanocatalyst has promising operational durability for repeated sonocatalytic applications (Figure 12a). Although the activity loss is not negligible, the catalyst still shows a relatively stable performance over multiple cycles, suggesting that the hybrid structure contributes to catalyst robustness and prevents rapid deactivation. Therefore, M-ZLT@Co_3_O_4_ can be considered a reusable and efficient sonocatalyst for the treatment of MG-contaminated wastewater [135].

#### 2.2.10. Effect of M-ZLT@Co_3_O_4_ Nanocatalyst on Different Dyes Contaminants

The degradation performance of the M-ZLT@Co_3_O_4_ nanocatalyst was evaluated toward different dye pollutants. This experiment is important because it demonstrates whether the catalyst is effective only for malachite green or whether it can be applied more broadly to structurally different organic dyes in wastewater. As shown in Figure 12b, the M-ZLT@Co_3_O_4_ nanocatalyst exhibited high sonocatalytic degradation efficiencies for all investigated dyes. The highest degradation efficiency was obtained for MG, reaching 99.31%, followed by CR with 95.78%, BY2 with 92.15%, MV with 90.24%, AO7 with 85.62%, and RB19 with 82.49%. This performance order can be expressed as, MG > CR > BY2 > MV > AO7 > RB19. The excellent degradation of MG confirms the strong affinity between the M-ZLT@Co_3_O_4_ surface and the target dye molecules under ultrasonic irradiation [136]. In addition, the high degradation efficiencies obtained for CR, MV, BY2, AO7, and RB19 indicate that the catalyst is not limited to a single dye molecule but can effectively promote the oxidative decomposition of various dye contaminants (Figure 12b). This broad-spectrum activity is highly valuable for real textile wastewater treatment, where multiple dye classes may coexist [137].

The differences in degradation efficiencies among the dyes can be attributed to their molecular structures, charge properties, chromophoric groups, steric effects, and interactions with the catalyst surface. Dyes with more favorable adsorption behavior on the CTAB-modified zeolite clay surface can be more easily concentrated near the active Co_3_O_4_ sites, thereby improving their exposure to reactive oxygen species generated during acoustic cavitation. Under ultrasonic irradiation, the collapse of cavitation bubbles produces localized high-temperature and high-pressure regions, leading to the formation of highly oxidative species such as •OH and •O_2_^−^ radicals. These reactive species attack chromophoric bonds, aromatic rings, and conjugated structures, resulting in dye decolorization and degradation [138]. The relatively lower degradation efficiencies observed for AO7 and RB19 may be related to their more complex molecular structures, stronger chromophoric stability, higher steric hindrance, or less favorable surface interaction with the catalyst. In particular, reactive and azo dyes often contain sulfonated groups and extended aromatic systems, which may reduce adsorption on the catalyst surface and increase resistance toward radical-mediated degradation. Nevertheless, even for these more resistant dyes, degradation efficiencies above 80% were achieved within 120 min, indicating that M-ZLT@Co_3_O_4_ possesses strong oxidative capability [139]. From a catalytic perspective, the high degradation performance can be associated with the synergistic role of the M-ZLT support and Co_3_O_4_ nanoparticles. The CTAB-modified zeolite clay matrix may enhance dye adsorption and improve the dispersion of Co_3_O_4_ active sites, while Co_3_O_4_ contributes to sonocatalytic activation and ROS generation. This combination facilitates efficient interfacial contact among dye molecules, catalyst surface, and sonochemically generated radicals [140].

#### 2.2.11. Degradation Effect of MG by Sonocatalytic Degradation Process of Different Catalysts

As shown in Figure 12c, the sonocatalytic performances of the different catalyst systems were systematically compared for MG degradation under identical experimental conditions over 120 min. The degradation efficiencies followed the order US/M-ZLT@Co_3_O_4_ (99.31%) > US/ZLT@Co_3_O_4_ (90.24%) > US/Co_3_O_4_ nanoparticles (78.48%) > US/M-ZLT (CTAB-ZLT) (75.63%) > US/ZLT (72.86%) > US/MG (35.71%). Among all investigated systems, M-ZLT@Co_3_O_4_ exhibited the highest sonocatalytic activity, achieving nearly complete MG degradation within 120 min. Importantly, the introduction of Co_3_O_4_ onto unmodified ZLT increased the degradation efficiency from 72.86% for ZLT to 90.24% for ZLT@Co_3_O_4_, demonstrating the substantial contribution of the Co_3_O_4_ active phase. Moreover, CTAB modification further increased the performance of ZLT@Co_3_O_4_ from 90.24% to 99.31% for M-ZLT@Co_3_O_4_. These comparative results provide direct experimental evidence that the outstanding activity of the final hybrid cannot be attributed exclusively to either Co_3_O_4_ or the zeolitic support, but rather arises from their effective integration together with the beneficial contribution of CTAB-mediated surface modification [141].

The relatively low MG degradation efficiency obtained with ultrasonic irradiation alone (US/MG, 35.71%) confirms that acoustic cavitation itself contributes to pollutant decomposition but is insufficient to achieve efficient removal under the investigated conditions. The formation, growth, and violent collapse of cavitation bubbles during ultrasonication generate highly localized regions of elevated temperature and pressure, facilitating the formation of reactive species, particularly hydroxyl radicals (•OH). However, in the absence of a solid catalyst, the generation and effective utilization of reactive species remain comparatively limited. The addition of pristine ZLT considerably increased the degradation efficiency to 72.86%, indicating that the zeolitic material contributes significantly to the overall process. This enhancement can primarily be associated with its porous structure and surface adsorption sites, which promote the accumulation of MG molecules at the solid–liquid interface and thereby increase their proximity to cavitation-derived reactive species. CTAB modification of ZLT resulted in a further increase in MG degradation from 72.86% to 75.63%. Although this improvement is relatively moderate in the absence of Co_3_O_4_, it demonstrates that surface modification contributes positively to the interaction between MG molecules and the zeolitic support, most likely by altering the surface environment and facilitating dye enrichment near accessible interfacial sites [142].

The Co_3_O_4_ nanoparticles exhibited a degradation efficiency of 78.48%, confirming their intrinsic contribution to the ultrasound-assisted catalytic oxidation of MG. Under ultrasonic irradiation, Co_3_O_4_ can facilitate interfacial redox processes and promote the formation of ROS. Nevertheless, the catalytic efficiency of unsupported Co_3_O_4_ nanoparticles may be constrained by particle aggregation and the consequent reduction in accessible surface-active sites. Immobilization of Co_3_O_4_ onto pristine ZLT markedly increased the degradation efficiency from 78.48% for unsupported Co_3_O_4_ to 90.24% for ZLT@Co_3_O_4_. This pronounced enhancement demonstrates the beneficial role of the ZLT support in improving the accessibility and dispersion of the Co_3_O_4_ phase while simultaneously providing adsorption sites capable of concentrating MG molecules in the vicinity of catalytically active centers. Thus, the ZLT@Co_3_O_4_ control provides important evidence that coupling the Co_3_O_4_ phase with the zeolitic matrix generates a more effective sonocatalytic system than either component used individually [143]. A particularly important observation is the further enhancement from 90.24% for unmodified ZLT@Co_3_O_4_ to 99.31% for CTAB-modified M-ZLT@Co_3_O_4_. This 9.07 percentage-point improvement clearly demonstrates the additional contribution of CTAB modification to the performance of the hybrid catalyst. The superior activity of M-ZLT@Co_3_O_4_ can therefore be interpreted in terms of several complementary effects. First, the M-ZLT matrix provides an effective support for Co_3_O_4_ nanoparticles, promoting their distribution over the zeolitic surface and reducing excessive particle aggregation, thereby preserving a greater number of accessible catalytic sites. Second, CTAB-mediated modification of the ZLT surface creates a more favorable interfacial environment for MG enrichment, increasing the local concentration of dye molecules in proximity to Co_3_O_4_ active centers. Third, intimate contact between the supported Co_3_O_4_ phase and M-ZLT may facilitate interfacial redox processes and improve the utilization of cavitation-derived reactive species during ultrasonic irradiation. The combined contribution of these effects provides a reasonable explanation for the substantially higher sonocatalytic activity of M-ZLT@Co_3_O_4_ compared with ZLT@Co_3_O_4_, Co_3_O_4_, M-ZLT, and pristine ZLT [144].

As a result, the control experiments reveal a progressive enhancement in MG degradation as the individual functions of ultrasound, ZLT, CTAB modification, and Co_3_O_4_ are integrated into the final hybrid system. The increase from 35.71% for US/MG to 72.86% for ZLT and 75.63% for M-ZLT demonstrates the contribution of the solid support and surface modification, whereas the increase to 78.48% for Co_3_O_4_ and 90.24% for ZLT@Co_3_O_4_ confirms the important catalytic role of Co_3_O_4_ and its beneficial interaction with the zeolitic support. Ultimately, the 99.31% degradation achieved by M-ZLT@Co_3_O_4_ demonstrates that CTAB modification and Co_3_O_4_ immobilization act cooperatively rather than independently. The resulting hybrid provides a favorable interfacial microenvironment in which MG molecules can be concentrated close to catalytically active sites and subsequently exposed to reactive species generated during acoustic cavitation, leading to highly efficient sonocatalytic degradation [145].

#### 2.2.12. Mechanistic Investigation of MG Degradation Products on M-ZLT@Co_3_O_4_ Under Sonocatalytic Process

As shown in Table 5, GC–MS analysis was carried out to identify the degradation by-products formed after 120 min of MG sonocatalytic treatment over the M-ZLT@Co_3_O_4_ nanocatalyst. The main detected intermediates included 6-indolizinecarboxamide, 2-methyl at 18.014 min with characteristic fragments of m/z 174, 130, 77, and 129; N,N-diethylacetamide at 15.332 min with fragments of m/z 58, 75, 73, and 30; trimethylsilanol benzoate at 26.901 min with fragments of m/z 105, 179, 77, and 135; tert-butyldimethylsilyloxy-substituted benzene at 17.562 min with fragments of m/z 207, 73, and 208; 1-methyl-2-dimethyl(isopropyl)silyloxycyclohexane at 12.049 min with fragments of m/z 75, 117, and 45; and di-TMS glyoxylic acid at 24.001 min with fragments of m/z 147, 75, and 73 (Table 5). The formation of these intermediates confirms that MG degradation proceeds through a multi-step oxidative pathway rather than simple adsorption or decolorization [146].

Under ultrasonic irradiation, acoustic cavitation induces the formation, growth, and violent collapse of microbubbles, generating highly reactive radical species such as •OH and •O_2_^−^. In the presence of M-ZLT@Co_3_O_4_, these species are generated more efficiently due to the catalytic contribution of Co_3_O_4_ and the adsorption-enhancing role of the CTAB-modified zeolite clay matrix. The MG molecules adsorbed on the catalyst surface are first subjected to radical attack on the chromophoric triphenylmethane structure, leading to disruption of the conjugated π-system and rapid decolorization [147]. Simultaneously, N-demethylation, dealkylation, and hydroxylation reactions may occur on the amino substituted aromatic rings. The detection of nitrogen-containing compounds such as 6-indolizinecarboxamide, 2-methyl and N,N-diethylacetamide suggests that the dimethylamino groups and nitrogen-bearing fragments of MG undergo oxidative rearrangement and fragmentation. Meanwhile, the presence of silylated aromatic compounds such as trimethylsilanol benzoate and tert-butyldimethylsilyloxy-substituted benzene indicates the formation of hydroxylated and carboxylated aromatic intermediates during the degradation process [148]. These aromatic intermediates can be further attacked by reactive oxygen species, resulting in ring opening reactions and the formation of smaller oxygenated molecules. The identification of di-TMS glyoxylic acid strongly supports the occurrence of deep oxidation and aromatic ring cleavage, since glyoxylic acid is a low-molecular-weight organic acid that can be formed after successive oxidative fragmentation of aromatic structures [149].

Therefore, the sonocatalytic degradation pathway of MG over M-ZLT@Co_3_O_4_ can be described as follows, adsorption of MG on the catalyst surface, generation of reactive oxygen species through ultrasound-induced cavitation and catalyst activation, radical attack on the chromophoric structure, N-demethylation and dealkylation of amino groups, cleavage of aromatic C–C/C–N bonds, formation of smaller aromatic and amide intermediates, ring opening reactions, generation of short chain organic acids such as glyoxylic acid, and final mineralization. These findings demonstrate that the M-ZLT@Co_3_O_4_ nanocatalyst promotes both efficient decolorization and substantial oxidative degradation of MG, highlighting its potential for the treatment of dye-contaminated wastewater [150].

#### 2.2.13. Proposed Sonocatalytic Degradation Mechanism of MG in Wastewater

**Figure 13:**
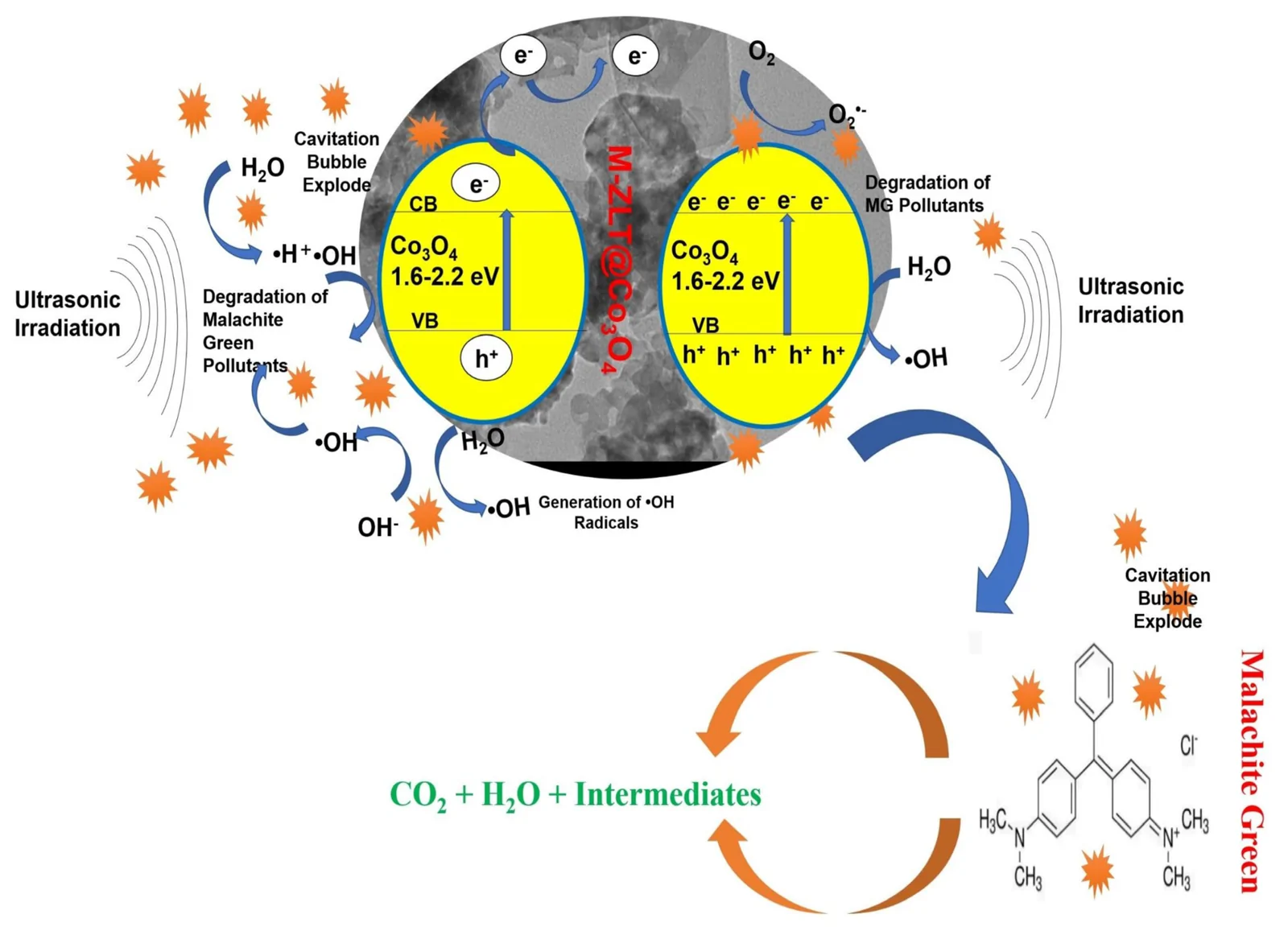
Possible degradation mechanism of MG from aqueous solutions by sonocatalytic degradation process.

As shown in Figure 13, the proposed sonocatalytic degradation mechanism of MG over the M-ZLT@Co_3_O_4_ nanocatalyst is mainly based on the synergistic interaction between ultrasonic cavitation and the catalytic activity of the Co_3_O_4_ loaded CTAB-modified zeolite clay structure. Under ultrasonic irradiation, acoustic cavitation produces transient hot spots through the rapid formation, growth, and collapse of microbubbles, generating highly energetic conditions in the aqueous medium. These localized environments activate the M-ZLT@Co_3_O_4_ surface and promote the generation of electron–hole pairs in Co_3_O_4_ [151]. The conduction-band electrons react with dissolved oxygen to produce superoxide radicals, whereas valence-band holes oxidize surface-adsorbed H_2_O or OH^−^ species to generate hydroxyl radicals. In parallel, the direct sonolysis of water molecules during cavitation further contributes to •OH radical formation. These reactive oxygen species subsequently attack the adsorbed MG molecules on the catalyst surface and in the bulk solution [152]. The superior sonocatalytic activity of M-ZLT@Co_3_O_4_ can be attributed to the multifunctional role of the hybrid structure. The M-ZLT support enhances MG adsorption and facilitates the accumulation of dye molecules near the active Co_3_O_4_ sites, while also improving the dispersion and stability of Co_3_O_4_ nanoparticles. This configuration increases the availability of active sites and promotes efficient interfacial charge transfer, thereby suppressing electron–hole recombination and improving ROS generation [153]. The superior sonocatalytic performance of M-ZLT@Co_3_O_4_ can be attributed to the cooperative functions of ultrasonic cavitation, the adsorption capability of the M-ZLT support and the catalytic activity of dispersed Co_3_O_4_ nanoparticles. Acoustic cavitation generates transient high-temperature and high-pressure micro environments together with intense interfacial phenomena that promote the formation of ROS through sonochemical reactions. The basic radical generation steps can be presented as follows in Equations (41)–(45).

*M-ZLT@Co*_3_*O*_4_ + *US → M-ZLT@Co*_3_*O*_4_ *(e^−^ + h^+^)* (41)

*H*_2_*O + US → •OH + •H* (42)

*e^−^ + O*_2_ → •*O*_2_^−^ (43)

*h*^+^ + *H*_2_*O/OH^−^ → •OH* (44)

*MG + •OH/•O*_2_^−^/*h^+^ → Degradation intermediates → CO*_2_ + *H*_2_*O* (45)

Although electronic excitation of Co_3_O_4_ and the subsequent formation of electron–hole (e^−^/h^+^) pairs under ultrasonic irradiation may additionally contribute to ROS generation, this pathway should be regarded as a possible mechanism rather than a directly demonstrated process in the present study, since no ultrasound-dependent spectroscopic measurement of charge carriers was performed. The scavenger experiments nevertheless provide indirect evidence for the participation of reactive radical species in MG degradation. Meanwhile, the M-ZLT matrix enhances the adsorption and local enrichment of MG molecules near the Co_3_O_4_ active sites and promotes the dispersion and interfacial stability of the oxide nanoparticles, thereby facilitating surface-mediated oxidation reactions. Thus, MG degradation over M-ZLT@Co_3_O_4_ is more appropriately described as a radical dominated sonocatalytic process governed primarily by cavitation induced sonochemistry and adsorption-assisted surface reactions, with ultrasound-induced electronic excitation of Co_3_O_4_ representing a plausible supplementary pathway rather than a conclusively established mechanism [154].

#### 2.2.14. Comparison of MG Sonocatalytic Degradation with Other Reported Methods

The sonocatalytic degradation efficiency of the M-ZLT@Co_3_O_4_ nanocatalyst was compared with previously reported systems for MG removal in order to evaluate the catalytic advantage of the present material. As shown in Table 6, M-ZLT@Co_3_O_4_ exhibited an excellent degradation efficiency of 99.31% toward MG under ultrasonic irradiation after 120 min. This high activity indicates that the hybrid catalyst is highly competitive with, and in some cases superior to, previously reported sonocatalysts and related advanced oxidation systems. The remarkable performance can be attributed to the synergistic contribution of Co_3_O_4_ nanoparticles and the CTAB-modified zeolite clay support. Co_3_O_4_ provides active semiconductor sites for ultrasound-assisted charge separation and reactive oxygen species generation, whereas M-ZLT enhances MG adsorption, improves Co_3_O_4_ dispersion, and facilitates interfacial contact between the pollutant molecules and catalytic active sites. Furthermore, the high degradation efficiency achieved under natural pH conditions and moderate catalyst dosage highlights the practical applicability of the proposed system. Although differences in experimental conditions such as dye concentration, catalyst loading, ultrasonic power, pH, and reaction time should be considered when comparing literature data, the present M-ZLT@Co_3_O_4_ system demonstrates outstanding sonocatalytic performance for MG degradation. These findings confirm that M-ZLT@Co_3_O_4_ is a promising, efficient, and reusable heterogeneous sonocatalyst for the treatment of dye containing wastewater.

#### 2.2.15. Quantitative Performance Comparison with Co_3_O_4_-Based Sonocatalysts

The quantitative comparison presented in Table 7 demonstrates that the M-ZLT@Co_3_O_4_ nanocatalyst exhibits highly competitive sonocatalytic performance relative to previously reported Co_3_O_4_-based catalytic systems. Under the optimized conditions of 20 mg L^−1^ MG, 1.0 g L^−1^ catalyst dosage, an ultrasonic power density of 300 W L^−1^ and a reaction time of 120 min, M-ZLT@Co_3_O_4_ achieved a degradation efficiency of 99.31%. This performance is comparable to or higher than those reported for several Co_3_O_4_-containing sonocatalysts, for which degradation efficiencies generally range from approximately 90% to 99%, depending strongly on catalyst composition, pollutant type, catalyst loading and ultrasonic operating conditions. For example, Co_3_O_4_/TiO_2_ required a considerably longer treatment period of approximately 420 min to achieve about 90% degradation, whereas, Fe@Co_3_O_4_ and C_3_N_4_-doped Fe@Co_3_O_4_ systems exhibited efficiencies of approximately 98–99% under their respective experimental conditions. Although some hybrid Co_3_O_4_ systems can provide similarly high or even complete removal, direct performance comparison should be interpreted cautiously because certain studies employ additional irradiation sources, oxidants, different ultrasonic frequencies/powers, or substantially different pollutant concentrations. Nevertheless, the 99.31% MG degradation obtained using M-ZLT@Co_3_O_4_ under ultrasound alone highlights the effectiveness of the hybrid architecture. The enhanced activity can reasonably be associated with the synergistic combination of efficient MG adsorption on the M-ZLT matrix, improved dispersion and accessibility of Co_3_O_4_ active sites, cavitation induced generation of reactive species, and intensified mass transfer at the catalyst solution interface. Accordingly, M-ZLT@Co_3_O_4_ represents a promising Co_3_O_4_-based sonocatalytic material for the treatment of dye-contaminated aqueous systems.

## 3. Experimental Section

### 3.1. Materials and Equipment

All chemicals used in this study were of analytical grade and were obtained from Merck (Darmstadt, Germany) and Sigma-Aldrich (Germany). Zeolite (ZLT), cetyltrimethylammonium bromide (CTAB), cobalt (II) chloride hexahydrate (CoCl_2_·6H_2_O), N,O-bis (trimethylsilyl)acetamide (BSA), malachite green (MG), sodium dihydrogen phosphate (NaH_2_PO_4_), sodium bicarbonate (NaHCO_3_), ethanol (C_2_H_6_O), ethylenediaminetetraacetic acid (EDTA, C_10_H_16_N_2_O_8_), chloroform (CHCl_3_), tert-butanol ((CH_3_)_3_COH), and p-benzoquinone (C_6_H_4_O_2_) were purchased from Sigma-Aldrich (Germany). Hydrochloric acid (HCl), sulfuric acid (H_2_SO_4_), sodium sulfate (Na_2_SO_4_), sodium chloride (NaCl), sodium fluoride (NaF), and sodium hydroxide (NaOH). Potassium iodide (KI) was also used as received from a commercial supplier without additional purification.

Distilled water was used throughout all synthesis, modification, and catalytic degradation experiments. All aqueous solutions were freshly prepared prior to use. The chemicals were utilized for zeolite modification, nanocatalyst synthesis, pH adjustment, radical scavenging experiments, and preparation of model pollutant solutions without any further treatment. For catalyst synthesis and modification in the liquid phase, mixing procedures were performed using a magnetic stirrer (IKA RCT Basic, Germany) under controlled stirring conditions. The drying of synthesized materials was carried out in a laboratory oven (Santen SE 125), while calcination processes were conducted in a programmable muffle furnace (Protech laboratory oven) at the desired temperatures. All chemicals and catalyst samples were accurately weighed using an analytical balance (Sartorius BP1106, Germany) with a precision of ±0.1 mg. The concentration of malachite green (MG) in the aqueous phase after sonocatalytic degradation was determined by UV–Vis spectrophotometry (PerkinElmer Lambda 25, UK) by monitoring the characteristic maximum absorption wavelength of the dye. The pH of the reaction solutions was adjusted using dilute HCl or NaOH solutions and measured with a benchtop pH meter (Hanna pH-2221). Following each degradation experiment, the catalyst particles were separated from the suspension by centrifugation using a BOECO S-8 centrifuge. Sonocatalytic degradation experiments were performed in an ultrasonic processor (WF-UD6 model sonicator) operating under controlled experimental conditions.

The structural, morphological, and physicochemical properties of the hydrothermally synthesized Modified-ZLT@Co_3_O_4_ and Co_3_O_4_ nanocatalysts were comprehensively characterized using advanced analytical techniques. Fourier-transform infrared spectroscopy (FT-IR, Bruker VERTEX 70v) was employed to identify surface functional groups and to elucidate possible interfacial interactions between Co_3_O_4_ and the modified zeolite framework. The crystalline structure and phase composition of the synthesized materials were analyzed by X-ray diffraction (XRD) using a PANalytical Empyrean diffractometer with Cu Kα radiation (λ = 1.5406 Å). The surface morphology and microstructural features were examined using scanning electron microscopy (SEM, Zeiss Sigma 300). Detailed nanoscale structural analysis was further performed by transmission electron microscopy (TEM, Hitachi High-Tech HT7700). Elemental composition and bulk chemical analysis were determined by X-ray fluorescence spectroscopy (XRF, Rigaku ZSX100e/100s). Textural properties, including specific surface area, pore volume, and pore size distribution, were evaluated by nitrogen adsorption–desorption measurements at 77 K using a Micromeritics 3Flex BET surface area analyzer. The specific surface area was calculated according to the Brunauer–Emmett–Teller (BET) method. In addition, intermediate oxidation products formed during the degradation process were identified by gas chromatography–mass spectrometry (GC–MS, Agilent Technologies 5975C-VL MSD), allowing elucidation of the possible degradation pathway and reaction mechanism.

### 3.2. Synthesis of Co_3_O_4_ Nanoparticles

Co_3_O_4_ nanoparticles were synthesized via a hydrothermal method. Briefly, 1.0 g of cobalt (II) chloride hexahydrate (CoCl_2_·6H_2_O) was dissolved in 20 mL of distilled water under continuous magnetic stirring until a homogeneous solution was obtained. The pH of the solution was then adjusted to 12.5 by the dropwise addition of 1.0 mol L^−1^ NaOH solution under vigorous stirring to promote complete precipitation of cobalt hydroxide precursors. The resulting suspension was stirred for 6 h at room temperature to ensure uniform nucleation and precipitation. Subsequently, the mixture was transferred into a Teflon-lined stainless-steel autoclave, sealed tightly, and subjected to hydrothermal treatment at 160 °C for 24 h. After naturally cooling to room temperature, the obtained precipitate was separated by filtration and thoroughly washed three to four times with distilled water to remove unreacted species and residual ions. The washed product was dried in a laboratory oven at 100 °C for 3 h and then calcined in a muffle furnace at 450 °C for 2 h in air to achieve phase transformation into crystalline Co_3_O_4_ nanoparticles. The final black powder was collected and stored in a desiccator for further characterization and catalytic experiments [164].

### 3.3. Synthesis of M-ZLT@Co_3_O_4_ Hybrid Nanocatalyst

The M-ZLT@Co_3_O_4_ hybrid nanocatalyst was synthesized through a controlled hydrothermal route followed by thermal treatment. Initially, 1.0 g of natural zeolite (ZLT) clay was dispersed in 100 mL of distilled water and magnetically stirred at room temperature for 24 h to promote adequate hydration, swelling, and accessibility of the exchangeable sites within the zeolitic structure. Subsequently, an aqueous cetyltrimethylammonium bromide (CTAB) solution, prepared at a concentration corresponding to the cation exchange capacity of the zeolite (1 CEC), was added dropwise to the ZLT suspension under continuous stirring. The resulting mixture was further stirred for 6 h to facilitate effective ion exchange, surfactant incorporation, and surface modification of ZLT, thereby producing the modified zeolite support (M-ZLT).

In a separate synthesis step, 1.0 g of CoCl_2_·6H_2_O was completely dissolved in 20 mL of distilled water under continuous magnetic stirring to obtain a homogeneous cobalt precursor solution. The pH of the solution was then gradually adjusted to 12.5 by dropwise addition of 1.0 mol L^−1^ NaOH solution, promoting the controlled precipitation of cobalt hydroxide precursor species. After reaching the desired pH, the suspension was continuously stirred for an additional 6 h to promote complete precipitation and homogeneous distribution of the cobalt-containing species. Thereafter, the previously prepared CTAB-modified zeolite suspension was gradually introduced into the cobalt-containing suspension under continuous stirring to ensure intimate contact between the M-ZLT support and cobalt precursor species.

The resulting homogeneous suspension was subsequently transferred into a Teflon-lined stainless-steel autoclave and tightly sealed. For the hydrothermal treatment, the autoclave was placed in a temperature-controlled laboratory oven, gradually heated from room temperature to 160 °C, and maintained isothermally at 160 °C for 24 h. This prolonged hydrothermal holding stage promoted nucleation, crystallization, and in situ deposition of cobalt-containing oxide precursor species onto and within the accessible surface regions of the modified zeolite framework. After completion of the 24 h hydrothermal treatment, the heating system was switched off and the autoclave was allowed to cool naturally to room temperature before opening, thereby avoiding abrupt thermal changes that could adversely affect the structural integrity of the resulting composite.

The recovered solid product was separated by filtration and repeatedly washed with distilled water to remove residual soluble ions, excess surfactant species, unreacted precursors, and other weakly bound impurities. The washed material was subsequently dried in a laboratory oven at 100 °C for 3 h to remove physically adsorbed and residual moisture. Following drying, the material was subjected to a controlled calcination procedure in a muffle furnace. The temperature was progressively increased from room temperature to 450 °C, 5 °C min^−1^ heating rate, after which the sample was maintained at 450 °C for 2 h.

**Figure 14:**
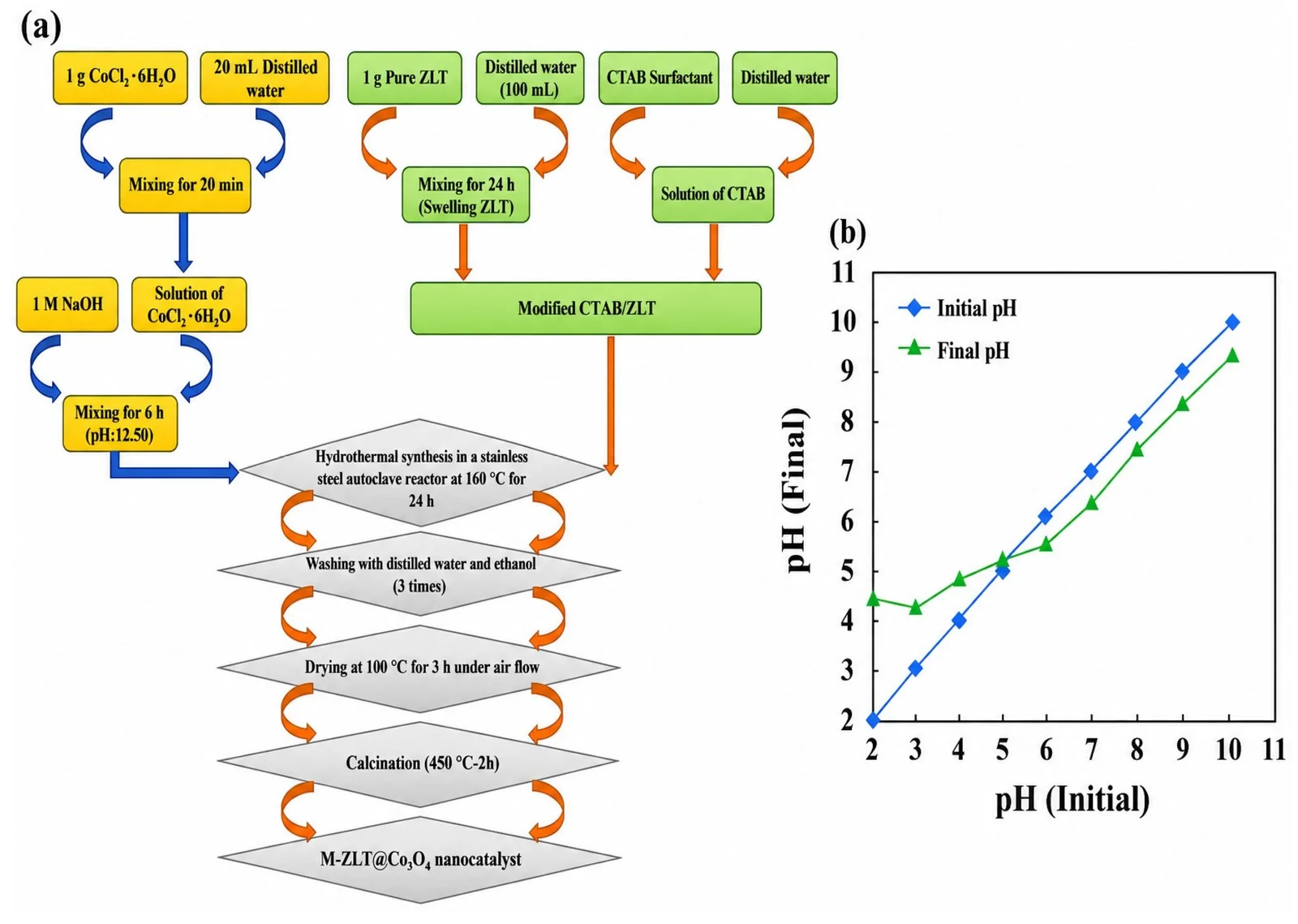
(**a**) Schematic representation of the hydrothermal synthesis process of the M-ZLT@Co_3_O_4_ hybrid nanocatalyst. (**b**) The point of zero charge (pHpzc) of the M-ZLT@Co_3_O_4_ hybrid nanocatalyst.

This thermal holding step facilitated decomposition of residual precursor species, removal of remaining organic surfactant fractions, conversion of the cobalt precursor into the crystalline Co_3_O_4_ phase, and stabilization of the Co_3_O_4_ species immobilized on the modified zeolite framework. After completion of calcination, the furnace was switched off and the material was allowed to cool gradually to room temperature inside the furnace to minimize thermally induced structural stress. The resulting crystalline M-ZLT@Co_3_O_4_ hybrid nanocatalyst was collected and stored in airtight containers under ambient conditions until subsequent physicochemical characterization and sonocatalytic degradation experiments were performed (Figure 14a) [165].

The point of zero charge (pHpzc) of the synthesized M-ZLT@Co_3_O_4_ nanocatalyst was determined using the pH drift method. A series of 0.01 mol L^−1^ NaCl solutions were prepared as background electrolyte solutions, and their initial pH values were adjusted within the range of 2–10 using 0.01 mol L^−1^ NaOH or 0.01 mol L^−1^ HNO_3_ solutions. For each experiment, 0.1 g of Modified-ZLT@Co_3_O_4_ nanocatalyst was added to 50 mL of NaCl solution with a predetermined initial pH in polyethylene centrifuge tubes. The suspensions were sealed and continuously stirred at room temperature for 24 h to allow sufficient equilibration between the solid surface and the solution. After reaching equilibrium, the suspensions were centrifuged to separate the solid phase, and the final pH values of the suspensions were measured using a calibrated pH meter. The difference between the initial and final pH values (ΔpH = pH_final_ − pH_initial_) was calculated for each sample. A plot of ΔpH versus initial pH was constructed, and the pH value at which ΔpH = 0 was taken as the pHpzc of the nanocatalyst. Based on this analysis, the pH_pzc_ of the M-ZLT@Co_3_O_4_ nanocatalyst was determined to be 5.40 (Figure 14b). Accordingly, the catalyst surface is positively charged at pH values below 5.40 and negatively charged at pH values above this value, which plays a critical role in electrostatic interactions during the sonocatalytic degradation process [166].

### 3.4. Experimental Procedure for Sonocatalytic Degradation

In the sonolytic and sonocatalytic degradation experiments, malachite green (MG) was selected as a model organic pollutant due to its widespread application in the textile, paper, and leather industries. MG is a cationic triphenylmethane dye that is frequently detected in industrial effluents and is known for its high chemical stability and persistence in aquatic environments. The chemical structures and some properties of the toxic dyes used in this study are given in Table 8.

**Figure 15:**
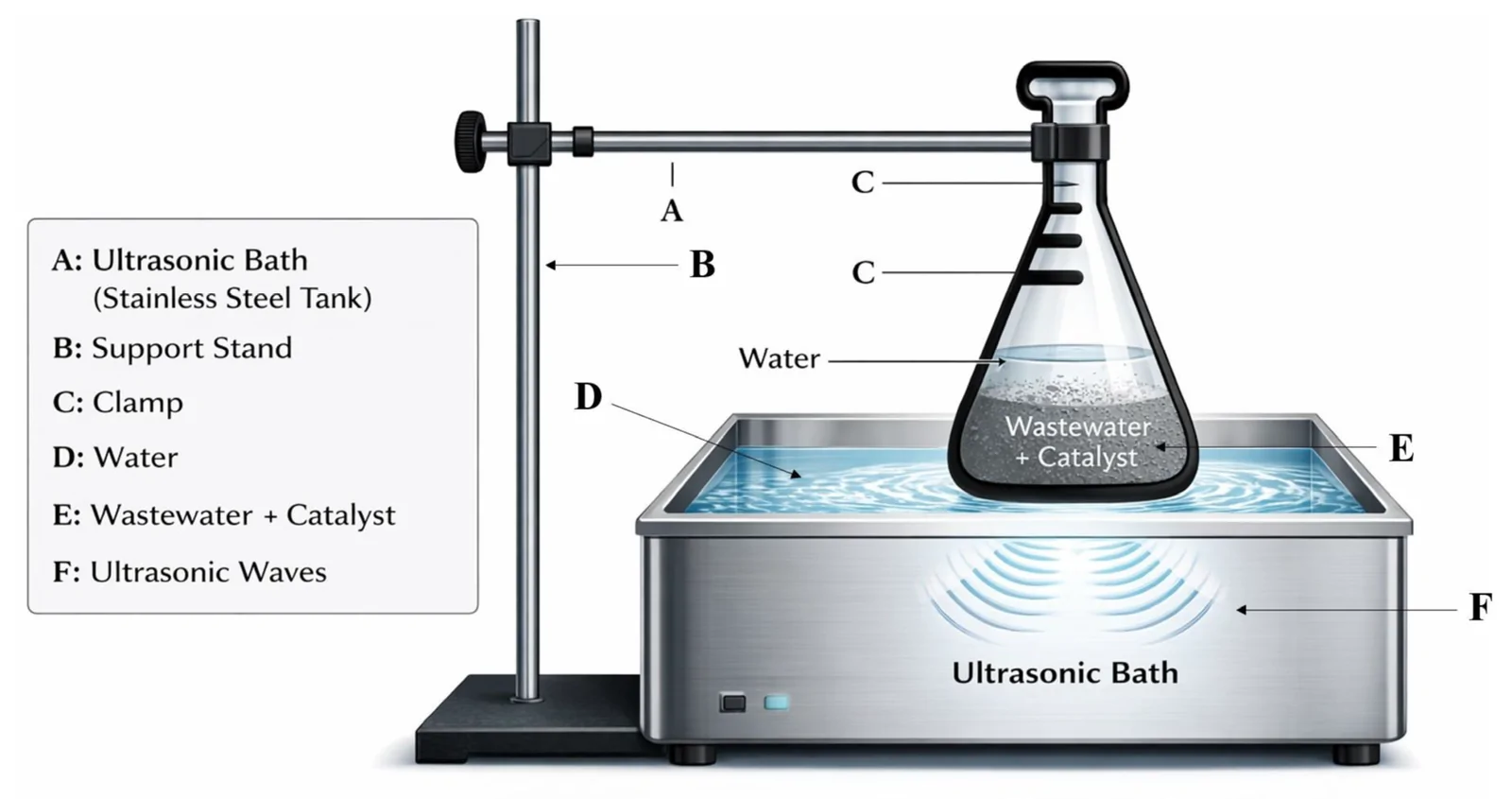
Schematic representation of the reactor used in sonocatalytic degradation experiments.

The sonocatalytic degradation of MG was performed using an ultrasonic processor (WF-UD6 model sonicator, Republic of Korea) operating under an air atmosphere. The ultrasonic system was operated at a fixed output power of 300 W and a working frequency of 60 kHz (Figure 15). In a typical experiment, 100 mL of MG aqueous solution with a predetermined initial concentration was placed in a reaction vessel, and the required amount of Modified-ZLT@Co_3_O_4_ nanocatalyst was added. The suspension was exposed to ultrasonic irradiation under natural pH conditions for a total reaction time of 120 min. At regular time intervals, aliquots were withdrawn from the reaction mixture and immediately centrifuged to remove catalyst particles. The residual concentration of MG in the solution was determined using a UV–Vis spectrophotometer (Perkin Elmer Lambda 25, UK) by monitoring the maximum absorption wavelength (λ_max_ = 617 nm). The degradation efficiency was calculated based on the relative decrease in MG concentration as a function of sonication time. The maximum absorption wavelength (λ_max_) of malachite green (MG) was experimentally determined to be 617 nm and was used for quantitative analysis throughout all degradation experiments. The sonocatalytic degradation performance of the Modified-ZLT@Co_3_O_4_ nanocatalyst was systematically evaluated under various operational parameters. These included the effect of initial dye concentration, catalyst dosage, and solution pH on degradation efficiency. In addition, the role of reactive species in the degradation mechanism was investigated using different inorganic and organic radical scavengers. Furthermore, the reusability and stability of the Modified-ZLT@Co_3_O_4_ nanocatalyst were assessed through consecutive sonocatalytic cycles under identical experimental conditions. The degradation efficiency (%) was calculated based on the relative decrease in MG concentration over time, enabling comprehensive evaluation of catalytic activity and operational stability. The dye degradation efficiencies were calculated using Equation (46).

*Degradation Efficiency (%) = (C*_0_ − *C_t_)/C*_0_ × 100 (46)

Here, C_0_ and C_t_ represent the initial concentration and the concentration at time t of the dye, respectively. For conducting the sonocatalytic oxidation of the MG dye, a WF-UD6 model sonicator was employed.

In the sonocatalytic oxidation system employing the Modified-ZLT@Co_3_O_4_ nanocatalyst, ultrasonic irradiation induces acoustic cavitation, resulting in the formation, growth, and implosive collapse of microbubbles within the liquid medium. The extreme local conditions generated during cavitation (high temperature and pressure) promote the excitation of the metal oxide structure, facilitating the generation of electron–hole (e^−^/h^+^) pairs. These charge carriers subsequently participate in redox reactions at the catalyst surface, leading to the enhanced formation of ROS, particularly hydroxyl radicals (•OH), in the aqueous phase. Furthermore, the introduction of an appropriate amount of heterogeneous or hybrid catalyst into the reaction system provides additional nucleation sites for cavitation bubble formation, especially at the solid–liquid interface. Surface irregularities and active sites on the catalyst act as preferential cavitation centers, accelerating bubble generation and collapse dynamics. This synergistic effect between ultrasonic cavitation and catalytic surface activity significantly increases •OH production, thereby improving the oxidative degradation efficiency of organic pollutants in solution.

### 3.5. Kinetic Study

The kinetic behavior was evaluated using the following equation derived from the simplified Langmuir–Hinshelwood model to Equation (47).

*−ln(C_t_/C*_0_*) = k*_app_ *t* (47)

where C_0_ and C_t_ represent the initial and time-dependent MG concentrations, respectively, and k_app_ (min^−1^) is the apparent pseudo-first-order rate constant obtained from the slope of the linear plots of −ln(C_t_/C_0_) versus irradiation time. The linearity of these plots for all investigated catalyst dosages confirms that the photocatalytic degradation of MG follows pseudo-first-order kinetics under the applied experimental conditions.

## 4. Conclusions

In this study, a novel M-ZLT@Co_3_O_4_ hybrid nanocatalyst was successfully fabricated through the hydrothermal immobilization of Co_3_O_4_ nanoparticles onto the surface of modified zeolite clay. XRF analysis showed that the hybrid material contained 49.26% Co_3_O_4_, confirming the effective incorporation of the Co_3_O_4_ phase into the ZLT-based structure. The structural and morphological properties of the synthesized materials were comprehensively evaluated using XRD, FT-IR, SEM/EDX, and TEM analyses. These characterization results verified the successful formation of the M-ZLT@Co_3_O_4_ hybrid nanocatalyst and confirmed that the applied hydrothermal strategy was suitable for obtaining a well-integrated composite structure. SEM and TEM observations demonstrated that Co_3_O_4_ nanoparticles were uniformly distributed and strongly anchored on the ZLT surface. In addition, BET surface area and BJH pore size distribution analyses indicated improved surface and pore characteristics compared with pure ZLT and Co_3_O_4_, which contributed positively to the enhanced sonocatalytic activity.

The sonocatalytic degradation performance of ZLT, Co_3_O_4_, and M-ZLT@Co_3_O_4_ was systematically investigated for MG removal from aqueous solution. All experiments were performed in triplicate, and the results were expressed as mean ± standard deviation to ensure reliability and reproducibility. Under optimized conditions of 1 g L^−1^ catalyst dosage and 20 mg L^−1^ MG concentration, M-ZLT@Co_3_O_4_ achieved 99.31% ± 0.45% degradation within 120 min. At the same catalyst dosage and 5 mg L^−1^ MG concentration, the degradation efficiency increased to 99.96% ± 0.64%, confirming the excellent catalytic efficiency of the hybrid material at lower pollutant loading. The solution pH strongly influenced the sonocatalytic degradation process. The degradation efficiency increased from 75.69% at pH 3 to 87.31% at pH 4, followed by markedly higher efficiencies of 99.31%, 99.42%, 98.93%, 99.45%, and 99.94% at natural pH 5.2, pH 6, pH 8, pH 10, and pH 11, respectively. These findings indicate that acidic conditions suppress MG degradation, whereas neutral and alkaline media promote the sonocatalytic oxidation process. The role of reactive species was evaluated using inorganic scavengers, including SO_4_^2−^, PO_4_^2−^, Cl^−^, F^−^, and I^−^, as well as organic scavengers such as chloroform, t-butanol, benzoquinone, and EDTA. Among the inorganic scavengers, chloride ions caused the greatest inhibition, decreasing the degradation efficiency to 72.65%. Among the organic scavengers, benzoquinone showed the strongest suppressive effect, reducing the efficiency to 79.97%. These results demonstrate that reactive oxygen species play a dominant role in the sonocatalytic degradation mechanism of MG.

The reusability of M-ZLT@Co_3_O_4_ was examined under constant conditions of 20 mg L^−1^ MG and 1 g L^−1^ catalyst dosage for six consecutive cycles. The degradation efficiency gradually decreased from 99.31% to 78.82%, indicating a moderate activity loss after repeated use. Nevertheless, the catalyst retained considerable performance, confirming its structural stability, operational durability, and potential for repeated wastewater treatment applications. From a practical viewpoint, the superior activity of M-ZLT@Co_3_O_4_ compared with the individual ZLT and Co_3_O_4_ components can be attributed to the synergistic interaction between Co_3_O_4_ nanoparticles and the modified ZLT support, together with improved surface area and pore properties. The high degradation efficiency obtained at natural pH 5.2 is particularly important because it eliminates the need for pH adjustment, thereby simplifying process operation and reducing chemical consumption. The influence of real water matrices followed the order distilled water (99.31%) > tap water (96.08%) > river water (88.84%) > lake water (86.90%). This trend confirms that the composition of the water matrix significantly affects the sonocatalytic performance. The almost complete MG degradation in distilled water suggests that, in the absence of interfering ions, natural organic matter, and suspended particles, the generated reactive oxygen species can more effectively attack dye molecules. Under ultrasonic irradiation, acoustic cavitation produces localized hot spots with high transient temperature and pressure, promoting water splitting and radical formation. Ultrasonic power was also identified as a critical operational parameter. After 120 min, the degradation efficiencies followed the order 500 W L^−1^ (99.90%) > 400 W L^−1^ (99.74%) > 300 W L^−1^ (99.31%) > 200 W L^−1^ (91.98%). This improvement with increasing ultrasonic power can be associated with stronger cavitation intensity, enhanced mass transfer, greater reactive oxygen species generation, and more effective activation of the catalyst surface.

The M-ZLT@Co_3_O_4_ nanocatalyst also exhibited high degradation ability toward different dye pollutants. The degradation efficiencies followed the order MG (99.31%) > CR (95.78%) > BY2 (92.15%) > MV (90.24%) > AO7 (85.62%) > RB19 (82.49%). These results confirm that the catalyst is not limited to MG degradation but can also promote the oxidative decomposition of various dye molecules with different structures. Comparative sonocatalytic tests further confirmed the superior performance of the hybrid catalyst, with degradation efficiencies following the order M-ZLT@Co_3_O_4_ nanocatalyst (99.31%) > Co_3_O_4_ nanoparticles (78.48%) > ZLT (72.86%) > US-MG alone (35.71%). The markedly higher efficiency of M-ZLT@Co_3_O_4_ demonstrates the strong synergistic effect between the CTAB-modified zeolite clay support and the Co_3_O_4_ active phase, resulting in improved surface interaction, radical formation, and catalytic activity.

The degradation kinetics were satisfactorily described by the pseudo-first-order kinetic model, with apparent rate constants of 0.0042–0.095 min^−1^ depending on catalyst dosage, 0.0046–0.0667 min^−1^ for different initial MG concentrations, and 0.0117–0.0620 min^−1^ under varying pH conditions. However, this kinetic interpretation should be considered as a simplified approximation of the Langmuir–Hinshelwood model. It is most applicable under dilute conditions, where MG adsorption on the catalyst surface remains limited. At higher MG concentrations, increased surface coverage, competition for active sites, and adsorption of degradation intermediates may reduce the strict validity of the Langmuir–Hinshelwood assumptions. As a result, M-ZLT@Co_3_O_4_ can be considered an efficient, reusable, and promising sonocatalyst for dye-contaminated wastewater treatment.

## Figures and Tables

**Table 1:** Surface area and porosity characteristics of pure ZLT, Co_3_O_4_ and M-ZLT@Co_3_O_4_ samples.

Samples	Pure ZLT	Co_3_O_4_	M-ZLT@Co_3_O_4_
BET surface area (m^2^ g^−1^)	22.0633	21.0466	28.0850
Langmuir surface area (m^2^ g^−1^)	332.1839	344.7713	496.8492
Micropore surface area (m^2^ g^−1^)	15.2497	28.7717	34.2621
Micropore volume (cm^3^ g^−1^)	0.0091	0.0159	0.0174
Total pore volume (Adsorption) (cm^3^ g^−1^)	0.0451	0.0519	0.0744
Total pore volume (Desorption) (cm^3^ g^−1^)	0.0549	0.0601	0.0892
Average pore diameter (Adsorption) (nm)	8.1609	9.8822	10.6015
Average pore diameter (Desorption) (nm)	9.9441	11.4336	12.7179
BJH average pore diameter (Adsorption) (nm)	14.0601	15.6729	17.3085
BJH average pore diameter (Desorption) (nm)	12.3035	16.4213	16.3602

**Table 2:** The chemical composition of the pure ZLT and M-ZLT@Co_3_O_4_ hybrid nanocatalyst.

Chemical Composition (%)	Co_3_O_4_	SiO_2_	Al_2_O_3_	CaO	K_2_O	MgO	Fe_2_O_3_	Na_2_O	Other *
Pure ZLT	-	71.86	13.22	2.76	2.61	2.28	1.41	0.18	5.69
M-ZLT@Co_3_O_4_	49.26	37.34	5.88	1.35	1.21	1.19	0.97	0.06	2.74

* Ba, Sr, Mn, Zr, S, and P in the form of their respective oxides.

**Table 3:** MG degradation rate constants with regression coefficients for parameters such as (**a**) catalyst dosage concentration, (**b**) initial MG concentration, (**c**) effect of pH and (**d**) effect of different process.

(a)		
Catalyst Dosage Concentration (g L^−1^)	k_app_ (min^−1^)	R^2^
0.1	0.0042	0.9873
0.2	0.0045	0.9639
0.4	0.0075	0.9790
0.6	0.0088	0.9680
0.8	0.0136	0.9567
1.0	0.0414	0.9325
1.5	0.0432	0.9806
2.0	0.0595	0.9830
**(b)**		
**Initial Dye Concentration (mg L^−1^)**	**k_app_ (min^−1^)**	**R^2^**
5	0.0667	0.9597
10	0.0502	0.9337
20	0.0414	0.9325
40	0.0163	0.9438
60	0.0114	0.9547
80	0.0089	0.9491
100	0.0067	0.9446
150	0.0046	0.9371
**(c)**		
**pH**	**k_app_ (min^−1^)**	**R^2^**
3	0.0117	0.9357
4	0.0172	0.9581
5.2	0.0414	0.9325
6	0.0430	0.9307
8	0.0378	0.9735
10	0.0435	0.9639
11	0.0620	0.9494
**(d)**		
**Different Process**	**k_app_ (min^−1^)**	**R^2^**
Sonocatalytic	0.0414	0.9325
Adsorption	0.0041	0.9589
Sonolytic	0.0036	0.9761

**Table 4:** Physicochemical characteristics of the real water matrices used in the sonocatalytic experiments.

Parameter	Unit	Tap Water	Lake Water	River Water
pH	–	7.52	8.09	7.88
Turbidity (NTU)	–	0.41	1.27	1.15
Electrical conductivity	μS cm^−1^	318	486	441
Total Hardness	mg L^−1^	12.8	68.4	57.6
Total dissolved solids	mg L^−1^	205	312	283
TOC	mg L^−1^	1.9	6.2	4.8
HCO_3_^−^	mg L^−1^	148	186	171
Cl^−^	mg L^−1^	19	17	23
SO_4_^2−^	mg L^−1^	31	38	43
NO_3_^−^	mg L^−1^	3.6	1.8	2.9
Ca^2+^	mg L^−1^	44	57	61
Mg^2+^	mg L^−1^	12	18	16
Na^+^	mg L^−1^	14	19	17
K^+^	mg L^−1^	2.1	3.1	2.7

**Table 5:** By-products identified based on GC-MS analysis after 120 min of reaction time during the sonocatalytic degradation process of MG.

No.	Compound Name	Structure	Retention Time (min)	Main Fragments
1	6-Indolizinecarboxamide, 2-methyl	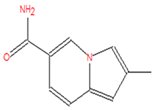	18.014	174 (100%), 130 (40%), 77 (14%), 129 (19%)
2	Acetamıde, N, N-Dıethyl-	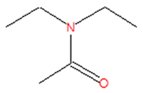	15.332	58 (100%), 75 (28%), 73 (25%), 30 (19%)
3	Silanol, Trimethyl-, Benzoate	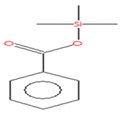	26.901	105 (100%), 179 (89%), 77 (80%), 135 (50%)
4	Benzene, 2-[(tert-butyldimethylsilyl) oxy]-1-isopropyl-4-methyl-	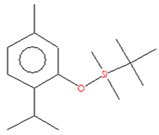	17.562	207 (100%), 73 (77%), 208 (19%)
5	1-Methyl-2-dimethyl(isopropyl) silyloxycyclohexane	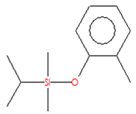	12.049	75 (100%), 117 (45%), 45 (17%)
6	Glyoxylic acid, di-TMS	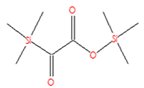	24.001	147 (100%), 75 (68%), 73 (62%)

**Table 6:** Comparison of catalytic activity of M-ZLT@Co_3_O_4_ nanocatalyst with some reported catalyst in the degradation of MG.

Catalyst	MG Concentration (mg L^−1^)	Catalyst Dosage (g L^−1^)	Degradation Efficiency (%)	Reaction Time (min)	Degradation Technique	Reference
Magnesium Ferrite	50	0.5	90.0	15	Sonocatalysis	[155]
NiGa_2_O_4_/CeO_2_	10	1.0	96.2	60	Sonocatalysis	[156]
Ag-ZnO	500	0.75	89.21	15	Sonocatalysis	[157]
Chitosan-Ascorbic Acid@NiFe_2_O_4_	70	1.0	99.05	90	Sonophotocatalysis	[158]
Ruthernium-Iridium	100	1.0	94.92	60	Ultrasonic-electrochemical	[78]
Zeolite Imidazole Framework-8	25	0.05	95.0	90	Sonocatalysis	[159]
M-ZLT@Co_3_O_4_	20	1.0	99.31	120	Sonocatalysis	This work

**Table 7:** Quantitative Performance Comparison with Co_3_O_4_-Based Sonocatalysts.

Catalyst	Target Pollutant	Catalyst Dosage (g L^−1^)	Degradation Efficiency (%)	Reaction Time (min)	Degradation Technique	Reference
Co_3_O_4_/TiO_2_	EDTA	2.0	≈90	420	Sonocatalysis	[160]
Fe@Co_3_O_4_	Methylene blue–Ciprofloxacin (MB–CF)	0.25	≈98	40–50	Sonocatalysis	[161]
C_3_N_4_-doped Fe@Co_3_O_4_	Methylene blue–Ciprofloxacin (MB–CF)	0.25	≈99	40–50	Sonocatalysis	[161]
Mesoporous Co_3_O_4_ NPs	Caffeine	0.20	22.7	55	Sonocatalysis	[162]
Co_3_O_4_/Fe^3+^ NPs	Amoxicillin	–	46.26	60	Sonocatalysis	[163]
M-ZLT@Co_3_O_4_	Malachite green	1.0	99.31	120	Sonocatalysis	This work

**Table 8:** Chemical structures and some properties of toxic dyes used in this study.

Name	Chemical Structure	Molecular Formula	M_w_ (g mol^−1^)	λ_max_ (nm)
Malachite Green (MG)	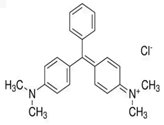	C_23_H_25_ClN_2_	463.50	617
Congo Red (CR)	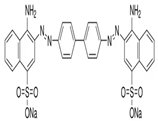	C_32_H_22_N_6_Na_2_O_6_S_2_	696.66	497
Methyl Violet (MV)	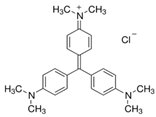	C_25_H_30_N_3_Cl	407.98	586
Acid Orange 7 (AO7)	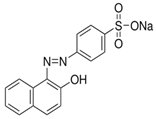	C_16_H_11_N_2_NaO_4_S	350.32	483
Reactive Blue 19 (RB19)	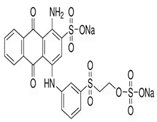	C_22_H_16_N_2_Na_2_O_11_S_3_	626.54	592
Basic Yellow 2 (BY2)	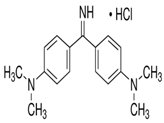	C_17_H_22_N_3_Cl	303.83	432

## Data Availability

Data will be made available on request.
